# Associations between the creatinine/cystatin C ratio and 28-day mortality in critically ill patients with sepsis: a retrospective cohort study

**DOI:** 10.3389/fnut.2026.1699579

**Published:** 2026-06-18

**Authors:** Cuiping Hao, Suzhen Zhang, Yuanqing Li, Anhao Liu, Qinghe Hu, Yaqing Zhang

**Affiliations:** 1 Department of Critical Care Medicine, Affiliated Hospital of Jining Medical University, Jining, Shangdong, China; 2 Department of Hematology, Affiliated Hospital of Jining Medical University, Jining, Shandong, China; 3 School of Nursing, Shandong First Medical University, Taian, Shandong, China; 4 Department of Neurology, Intensive Care Unit, Affiliated Hospital of Jining Medical University, Jining, Shandong, China

**Keywords:** 28-day mortality, creatinine/cystatin C ratio, critically ill patients, ICU, sepsis

## Abstract

**Background:**

The creatinine/cystatin C ratio (Cr/CysC ratio) has been correlated with muscle mass and survival in several patient populations. However, its relationship with sepsis prognosis remains underexplored. This study investigated the association between the Cr/CysC ratio and 28-day mortality in critically ill patients with sepsis.

**Methods:**

In this retrospective cohort study, we analyzed single-center clinical data from a tertiary general hospital in China to investigate 28-day mortality among patients with sepsis. We employed Cox proportional hazards models, Kaplan–Meier survival curves, and restricted cubic spline regression to evaluate the association between the creatinine-to-cystatin C (Cr/CysC) ratio and 28-day mortality. Additionally, subgroup and comprehensive sensitivity analyses were conducted to verify the robustness of our findings.

**Results:**

In total, 1,103 patients with sepsis were included, 46.3% of whom were male; moreover, the 28-day mortality rate was 29.9%. After adjustments for potential confounders, an independent relationship was observed between the Cr/CysC ratio and 28-day mortality via multivariable Cox regression analysis (HR: 0.39; 95% CI: 0.24–0.63; *p* < 0.001). When the data were stratified into quartiles, the risk of 28-day mortality was significantly lower in the highest quartile (HR: 0.44; 95% CI: 0.31–0.64; *p* < 0.001) than in the lowest quartile. RCS analysis showed an inverse association between Cr/CysC ratio and 28-day mortality. Sensitivity analyses yielded consistent results.

**Conclusion:**

In critically ill patients with sepsis, the Cr/CysC ratio on ICU admission was significantly associated with 28-day mortality. However, further validation in larger prospective studies is needed.

## 1 Background

Sepsis is defined as life-threatening organ dysfunction caused by a dysregulated host response to infection (1, 2). It is a leading cause of mortality among ICU patients (3), with reported mortality rates of 20–30%, increasing to 40–50% when complicated by respiratory or circulatory failure (4–6). Sarcopenia is defined as decreased muscle mass, strength, and physical function (7). Recent studies have demonstrated that skeletal sarcopenia affects the clinical outcomes of critically ill patients, including those with sepsis (8–12). In most studies, sarcopenia is diagnosed by measuring the skeletal muscle area (SMA) using computed tomography (CT) or ultrasound (13, 14). However, this approach requires patients to undergo additional imaging procedures.

In recent years, numerous researchers have adopted the “sarcopenia index” (SI), which is based on the serum creatinine/cystatin C ratio (Cr/CysC ratio), as a novel and reliable method for estimating muscle mass (15, 16). Serum creatinine (Cr) is a primary clinical indicator for assessing the glomerular filtration rate (GFR) and overall kidney function (17, 18). Cystatin C, which is a 13.4 kDa serine protease inhibitor, is synthesized by all nucleated cells and is cleared from the circulation through glomerular filtration (19). Recent studies have demonstrated that cystatin C and derived measures of renal function are risk factors for acute kidney injury (AKI) and mortality in patients with sepsis (20). Moreover, serum cystatin and allantoin levels are reportedly useful for predicting the development of AKI in elderly patients with sepsis (21). Other studies have demonstrated that the admission Cr/CysC ratio is associated with prognosis in patients with heart failure, chronic obstructive pulmonary disease, and several types of cancer (22–25).

In critically ill patients in the ICU, the combination of cystatin C and serum creatinine can be used to assess muscle mass, and these indicators are associated with malnutrition and poor ICU outcomes (26). Serum cystatin C exhibits greater predictive value than creatinine for prognosis in ICU patients discharged to the ward (27). Additionally, an elevated Cr/CysC ratio has been associated with improved survival in patients receiving intensive care and continuous kidney replacement therapy (CKRT) (28). However, the relationship between the serum Cr/CysC ratio and mortality in ICU patients with sepsis remains unclear. Therefore, this study aimed to explore the association between the Cr/CysC ratio and 28-day mortality in a large cohort of critically ill patients with sepsis.

## 2 Methods

### 2.1 Study population and design

**Figure 1:**
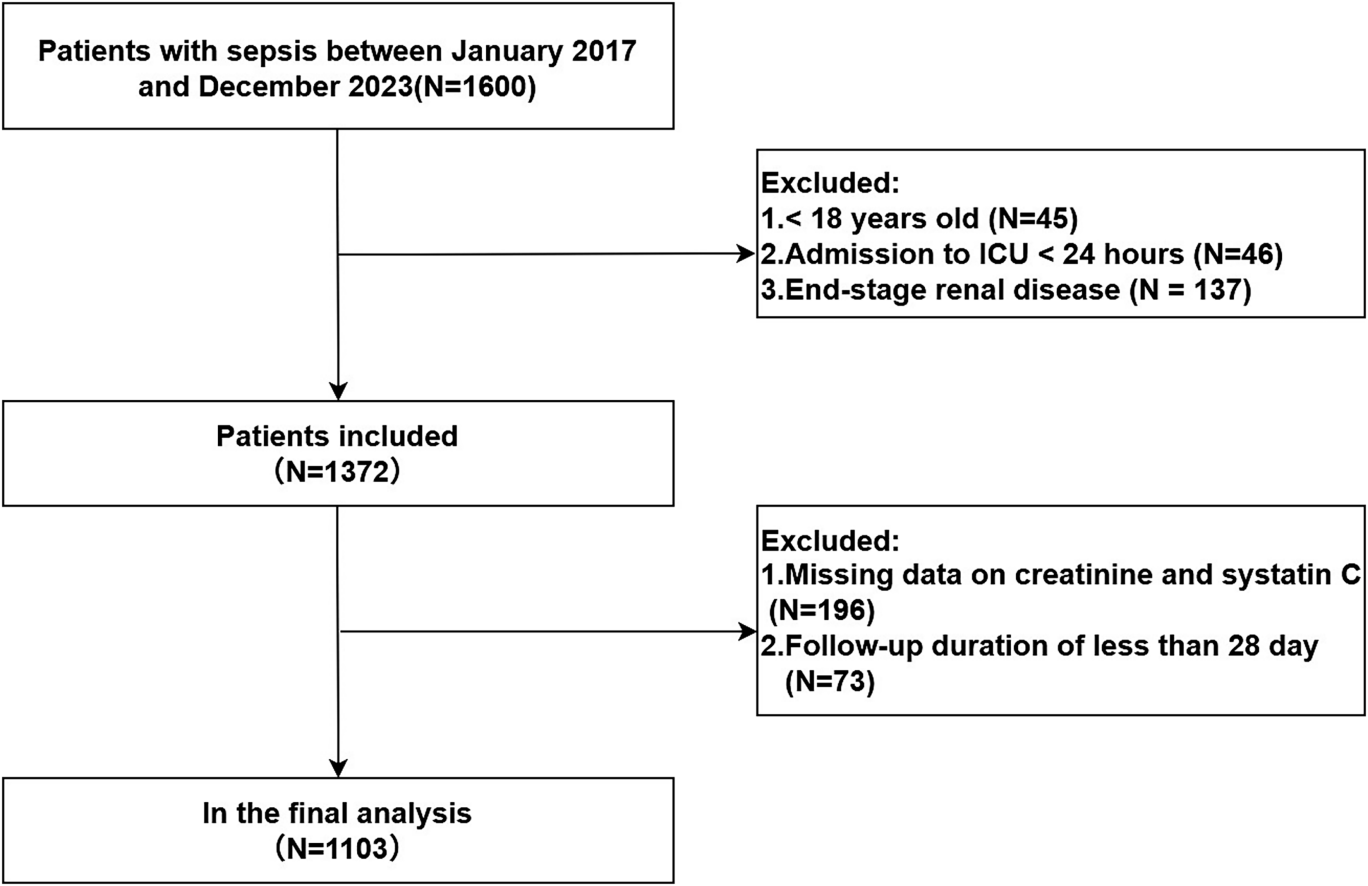
Flowchart of the study cohort.

In this retrospective cohort study, we screened critically ill patients with sepsis admitted to the intensive care unit (ICU) at the Affiliated Hospital of Jining Medical University (a tertiary general hospital in China) between January 2017 and December 2023. Sepsis was defined according to the Third International Consensus Definitions for Sepsis and Septic Shock (Sepsis-3) (2). Patients with suspicion of infection and a Sequential Organ Failure Assessment (SOFA) score ≥ 2 within the first 24 h of ICU stay were initially included (29). The following criteria were used to further exclude patients from this study: (1) age of less than 18 years; (2) patients who stayed in the ICU for less than 24 h; (3) end-stage kidney disease; (4) lacking data of creatinine and cystatin C level; (5)follow-up duration < 28 days (Figure 1).

This study was approved by the Ethics Committee of the Affiliated Hospital of Jining Medical University (approval number: 2023-11-C032). Given the retrospective nature of the study and the use of anonymized patient data extracted from institutional databases, the requirement for informed consent was waived by the Ethics Committee. All methods were performed in accordance with relevant guidelines and regulations.

### 2.2 Data collection

Clinical data for patients with sepsis were retrieved from the hospital’s electronic medical record system. We extracted information on patient demographics, laboratory parameters, treatments, comorbidities, and outcomes, as detailed in our previous publication (30). Severity scores included the Sequential Organ Failure Assessment (SOFA) and Acute Physiology and Chronic Health Evaluation II (APACHE II) scores. All aforementioned data were collected from the first available measurements within 24 h of ICU admission. The creatinine-to-cystatin C (Cr/CysC) ratio was calculated using concomitantly measured baseline values of serum creatinine (mg/dL) and cystatin C (mg/L). The estimated glomerular filtration rate (eGFR) was estimated using the 2021CKD-EPI creatinine equation (31), which incorporates sex, age and serum creatinine (Scr, mg/dL). The equation used was:


$$\begin{array}{c} \text{eGFR}=142\times \min{\left(\mathrm{Scr}/\kappa ,1\right)}^{\alpha }\times \max{\left(\mathrm{Scr}/\kappa ,1\right)}^{-1.200}\\ \times 0.9938^{\mathrm{Age}}\times 1.012\;[\text{if female}] \end{array}$$

where κ = 0.7 for females and 0.9 for males, and α = −0.241 for females and −0.302 for males. Scr is expressed in mg/dL.

AKI was diagnosed according to the Kidney Disease: Improving Global Outcomes (KDIGO) consensus criteria (32). This study included patients who developed AKI within 7 days of ICU admission.

### 2.3 Main results

The primary outcome was 28-day mortality. Survival data were retrieved from inpatient and outpatient electronic medical records. Time zero was defined as the date of ICU admission, with follow-up censored at 28 days. For patients who died within 28 days, survival time was calculated from ICU admission to the date of death. Patients known to be alive at 28 days (including those discharged alive after day 28) were administratively censored at day 28. For patients discharged alive before day 28, post-discharge vital status was ascertained from outpatient records or other documented contacts, and survival time was calculated to the date of last contact (or death). Patients with no documented contact within the 28-day window and whose vital status at day 28 could not be determined were considered lost to follow-up and excluded from the survival analysis.

### 2.4 Statistical analysis

The data were classified into four groups based on quartiles of the Cr/CysC ratio (Q1: 0.05–0.46, Q2: 0.47–0.61, Q3: 0.62–0.78, and Q4: 0.79–3.46). Categorical variables are expressed as numbers and percentages. Continuous variables are expressed as means and standard deviations (SDs) for normally distributed data or medians and interquartile ranges (IQRs) for skewed data. The chi-square test, one-way ANOVA, and Kruskal-Wallis test were used to compare categorical, normally distributed, and non-normally distributed continuous variables, respectively. For all variables, the percentage of missing data was less than 10% (Supplementary Table S1). To appropriately handle these missing data, multiple imputation was performed using the chained equations approach with five imputations in the R mice package to maximize statistical power and minimize bias.

Before analysis, the proportional hazards assumption was assessed using Schoenfeld residuals, which showed no significant violation (*p* > 0.05; Supplementary Table S2). Multivariable Cox regression analyses were performed to evaluate the associations between the Cr/CysC ratio and 28-day mortality. Multicollinearity was assessed using the variance inflation factor (VIF); a VIF ≥ 5 was considered indicative of multicollinearity. No multicollinearity was detected among the variables (Supplementary Table S3).

Six models were fitted in the Cox regression analysis; Model 1 was unadjusted. The Kaplan–Meier method was used to plot survival curves, and differences in survival among the four Cr/CysC ratio groups were compared using the log-rank test. Restricted cubic spline regression was employed to examine the dose–response relationship between the Cr/CysC ratio and 28-day mortality. Stratified analyses were further conducted according to age, sex, BMI, APACHE II score, and SOFA score to evaluate the robustness of the Cr/CysC ratio in predicting 28-day mortality. The likelihood ratio test was used to examine interactions between the Cr/CysC ratio and stratification variables. We also assessed the robustness of this association to potential unmeasured confounding by calculating E-values (33). The net reclassification improvement (NRI) and integrated discrimination improvement (IDI) were employed to further evaluate the incremental predictive utility of the Cr/CysC ratio beyond the APACHE II and SOFA scores.

All the analyses were performed with the statistical software packages R 4.2.2 (http://www.R-project.org, The R Foundation) and Free Statistics software versions 2.2 (Version 2.2, Beijing, China, http://www.clinicalscientists.cn/freestatistics) (34). A two-tailed test was conducted in our study, and a value of *p* < 0.05 was considered to be statistically significant.

## 3 Results

### 3.1 General characteristics of the participants

This study included 1,103 critically ill patients with sepsis based on specific inclusion and exclusion criteria. The baseline clinical characteristics are summarized in Table 1. The mean age was 67.1 ± 15.8 years, and 511 patients (46.3%) were male. The median baseline creatinine and cystatin C levels were 0.9 (IQR: 0.7–1.4) mg/dL and 1.5 (IQR: 1.2–2.1) mg/L, respectively, with Cr/CysC ratio ranging from 0.05 to 3.46. When patients were categorized into quartiles according to the Cr/CysC ratio (Q1, Q2, Q3, and Q4), those in the higher quartile groups demonstrated greater BMI and younger age (*p* < 0.05). Moreover, eGFR (*p* < 0.001) and serum cystatin C levels (*p* < 0.001) were lower in the higher Cr/CysC ratio quartiles.

**Table 1:** Baseline characteristics of critical patients with sepsis grouped according to Cr/CysC ratio quartiles.

Variables	Total (n = 1,103)	Q1 (0.05–0.46) (*n* = 276)	Q2 (0.47–0.61) (*n* = 275)	Q3 (0.62–0.78) (*n* = 276)	Q4 (0.79–3.46) (*n* = 276)	*p-*value
Demographics						
Age(years)	67.1 ± 15.8	68.3 ± 16.0	69.9 ± 15.5	66.4 ± 15.8	63.6 ± 15.2	< 0.001
Male (%)	511 (46.3)	146 (52.9)	125 (45.5)	115 (41.7)	125 (45.3)	0.06
BMI (Kg/m^2^)	22.7 ± 4.4	22.2 ± 4.4	22.6 ± 4.6	22.8 ± 4.0	23.4 ± 4.3	0.023
Smoking (%)	376 (34.1)	84 (30.4)	92 (33.5)	105 (38.2)	95 (34.4)	0.29
Drinking (%)	355 (32.2)	87 (31.5)	84 (30.5)	88 (32)	96 (34.8)	0.741
Admission year, *n* (%)						0.058
2017–2019	267 (24.2)	72 (26.1)	63 (22.9)	53 (19.2)	79 (28.6)	
2020–2023	836 (75.8)	204 (73.9)	212 (77.1)	223 (80.8)	197 (71.4)	
Infection (%)						< 0.001
Blood	44 (4.0)	12 (4.4)	10 (3.6)	12 (4.3)	10 (3.6)	
Lung	505 (45.8)	141 (51.3)	143 (52)	110 (39.9)	111 (40.2)	
Abdominal	329 (29.9)	53 (19.3)	74 (26.9)	98 (35.5)	104 (37.7)	
Urinary tract	144 (13.1)	36 (13.1)	32 (11.6)	41 (14.9)	35 (12.7)	
Skin or soft tissue	80 (7.3)	33 (12)	16 (5.8)	15 (5.4)	16 (5.8)	
Vital Signs (%)						
Heart rate (beats/min)	107.3 ± 24.8	106.8 ± 24.1	107.2 ± 24.8	107.3 ± 27.4	108.1 ± 22.6	0.945
MAP (mmHg)	82.9 ± 19.8	82.8 ± 17.7	85.0 ± 21.0	81.7 ± 21.1	82.0 ± 19.2	0.199
Respiratory rate(beats/min)	24.2 ± 7.2	24.3 ± 6.7	24.5 ± 7.3	23.7 ± 7.4	24.5 ± 7.3	0.546
Temperature(◦C)	37.5 ± 3.3	37.3 ± 1.0	37.3 ± 1.0	37.5 ± 1.0	37.9 ± 6.2	0.259
PAO_2_/FIO_2_	233.0 (171.0, 340.0)	224.0 (160.0, 307.2)	240.0 (183.0, 327.0)	264.0 (174.5, 355.0)	224.0 (167.5, 346.0)	0.054
Comorbidities (%)						
Hypertension	418 (37.9)	112 (40.6)	113 (41.1)	100 (36.2)	93 (33.7)	0.219
Coronary heart	298 (27.1)	78 (28.4)	77 (28)	82 (29.7)	61 (22.2)	0.199
Diabetes	293 (26.6)	76 (27.5)	70 (25.5)	78 (28.3)	69 (25)	0.794
Arrhythmia	296 (26.9)	82 (29.7)	76 (27.7)	79 (28.6)	59 (21.5)	0.123
Stroke	277 (25.1)	85 (30.9)	74 (26.9)	63 (22.8)	55 (19.9)	0.018
COPD	100 (9.1)	22 (8)	24 (8.7)	34 (12.3)	20 (7.2)	0.165
Liver disease	119 (10.8)	29 (10.5)	31 (11.3)	29 (10.5)	30 (10.9)	0.989
Cancer	110 (10.0)	26 (9.4)	25 (9.1)	33 (12)	26 (9.4)	0.656
Interventions						
Mechanical Ventilation (%)	622 (56.4)	156 (56.5)	148 (53.8)	152 (55.1)	166 (60.1)	0.472
Vasoactive drug use (%)	727 (65.9)	189 (68.5)	173 (62.9)	168 (60.9)	197 (71.4)	0.033
APACHEII	19.4 ± 8.1	20.2 ± 7.6	19.5 ± 8.1	18.9 ± 8.3	19.2 ± 8.3	0.272
SOFA	8.5 ± 3.6	8.9 ± 3.9	8.5 ± 3.5	8.3 ± 3.8	8.5 ± 3.3	0.295
Laboratory variables						
LAC (mmol/L)	2.0 (1.3, 3.5)	2.0 (1.2, 3.3)	1.8 (1.3, 2.8)	1.9 (1.3, 3.5)	2.3 (1.5, 4.5)	< 0.001
PLT (×10^9^ /L)	163.0 (89.5, 253.0)	166.5 (91.0, 256.0)	165.0 (87.0, 261.0)	160.0 (93.5, 246.5)	163.5 (88.0, 247.8)	0.978
PCT (ng/mL)	5.1 (0.6, 29.2)	2.1 (0.4, 12.6)	4.1 (0.4, 20.5)	7.3 (1.0, 31.0)	14.8 (1.7, 67.7)	< 0.001
ALB (g/L)	29.1 (25.1, 33.1)	28.1 (24.5, 31.9)	29.0 (25.0, 32.8)	29.3 (25.5, 33.3)	30.1 (26.1, 34.3)	0.001
PT (s)	14.5 (13.0, 16.5)	14.4 (13.0, 16.3)	14.4 (12.9, 16.7)	14.6 (13.1, 16.5)	14.6 (13.0, 16.4)	0.689
APTT (s)	34.5 (29.9, 40.0)	35.0 (30.2, 41.2)	35.0 (29.9, 40.8)	33.9 (29.1, 39.6)	34.4 (30.4, 39.5)	0.402
TBIL (umol/L)	17.0 (10.8, 30.3)	16.4 (10.4, 26.5)	16.4 (11.0, 29.8)	17.8 (10.9, 30.8)	18.4 (11.8, 33.4)	0.068
WBC (×10^9^ /L)	11.2 (7.0, 16.6)	10.8 (6.6, 15.9)	11.6 (7.3, 17.4)	11.5 (7.8, 16.8)	11.1 (6.6, 16.2)	0.188
ALT (U/L)	27.3 (15.6, 58.3)	22.7 (13.2, 50.0)	26.6 (15.9, 51.3)	29.4 (15.9, 58.0)	31.6 (17.8, 70.0)	0.001
CRP (mg/L)	111.0 (39.9, 180.0)	92.7 (40.0, 167.3)	115.8 (38.5, 180.3)	106.7 (41.2, 178.9)	123.0 (41.3, 187.5)	0.092
Creatinine (mg/L)	0.9 (0.7, 1.4)	0.6 (0.4, 0.9)	0.8 (0.6, 1.2)	1.0 (0.8, 1.3)	1.4 (1.0, 1.8)	< 0.001
Cystatin C(mg/L)	1.5 (1.2, 2.1)	1.8 (1.3, 2.7)	1.6 (1.2, 2.2)	1.5 (1.2, 2.0)	1.3 (1.0, 1.8)	< 0.001
BUN (mmol/L)	8.9 (5.8, 13.7)	7.1 (4.6, 11.5)	8.5 (5.5, 13.1)	9.3 (6.4, 13.3)	11.0 (7.8, 15.4)	< 0.001
Uric acid (umol/L)	268.0 (189.5, 365.0)	239.0 (136.0, 345.0)	265.0 (181.0, 347.0)	272.0 (204.5, 375.5)	297.0 (222.0, 389.0)	< 0.001
β_2_-MG (mg/L)	3.8 (2.6, 6.0)	4.1 (2.7, 7.9)	4.0 (2.8, 6.7)	3.6 (2.6, 5.5)	3.5 (2.3, 5.0)	< 0.001
eGFR (mL/min/1.73m^2^)	85.3 (55.5, 104.3)	103.7 (84.3, 117.3)	90.0 (65.9, 103.5)	81.4 (54.7, 98.1)	58.5 (42.4, 87.6)	< 0.001
Outcome						
ICU Los(day)	6.0 (3.0, 12.0)	7.0 (3.0, 14.0)	6.0 (3.0, 13.0)	6.0 (3.0, 11.0)	5.0 (3.0, 10.2)	0.011
Hospital Los(day)	11.0 (6.0, 19.0)	11.0 (5.0, 22.2)	11.0 (6.0, 21.0)	11.0 (6.0, 17.0)	11.5 (6.0, 19.0)	0.888
AKI (%)	255 (23.1)	59 (21.4)	57 (20.7)	60 (21.7)	79 (28.6)	0.095
CRRT (%)	173 (15.7)	30 (10.9)	41 (14.9)	38 (13.8)	64 (23.2)	< 0.001
Hospital mortality (%)	386 (35.0)	139 (50.4)	94 (34.2)	85 (30.8)	68 (24.6)	< 0.001
28-day mortality (%)	330 (29.9)	114 (41.3)	78 (28.4)	74 (26.8)	64 (23.2)	< 0.001

Data conforming to a normal distribution are presented as mean ± standard deviation, while non-normally distributed data are displayed as median along with interquartile range; numbers (proportions) for categorical variables.

BMI, body mass index; SOFA, sequential organ failure assessment score; APACHE II, Acute Physiology and Chronic Health Score II; LAC, Lactic acid; MAP, mean blood pressure; COPD, chronic obstructive pulmonary disease; WBC, white blood cell; PLT, platelets; PCT, procalcitonin; ALT, alanine aminotransferase; ALB, albumin; TBIL, total bilirubin; PT, prothrombin time; APTT, activated partial thromboplastin time; BUN, blood urea nitrogen; AKI, acute kidney injury; β2-MG, β2-microglobulin; LOS, length of stay; ICU, intensive care unit.

AKI was observed in 255 (23.1%) patients, and the rate of CRRT dependence was 15.7%. The 28-day mortality rate was 29.9%. Additionally, the mean lengths of ICU and hospital stays were 6.0 (IQR: 3.0–12.0) and 11.0 (IQR: 6.0–19.0) days, respectively. The trend toward lower 28-day mortality at a higher Cr/CysC ratio was highly significant (*p* < 0.001) (Table 1).

### 3.2 Association between Cr/CysC ratio and 28-day mortality

After performing univariate Cox regression analyses (Supplementary Table S4), we constructed six multivariable Cox regression models to assess the relationship between the Cr/CysC ratio and 28-day mortality risk in patients with sepsis. In the fully adjusted model, the Cr/CysC ratio remained significantly associated with 28-day mortality (Table 2). For each unit increase in the Cr/CysC ratio, the risk of 28-day mortality decreased by 59% (HR: 0.41; 95% CI: 0.27–0.63; *p* < 0.001). When analyzed as quartiles with Q1 as the reference, the risk of mortality in Q4 decreased by 56% after adjusting for confounders (HR: 0.44; 95% CI: 0.31–0.64; Table 2, Model 6).

**Table 2:** Multivariate COX regression analyses of Cr/CysC ratio and incidence of 28-day mortality.

Variable	Model 1		Model 2		Model 3		Model 4		Model 6		Model 6	
	HR (95%CI)	*p*	HR (95%CI)	*p*	HR (95%CI)	*p*	HR (95%CI)	*p*	HR (95%CI)	*p*	HR (95%CI)	*p*
^a^Cr/CysC ratio	0.41 (0.27 ~ 0.63)	<0.001	0.46 (0.3 ~ 0.71)	<0.001	0.52 (0.34 ~ 0.81)	0.004	0.45 (0.29 ~ 0.69)	<0.001	0.39 (0.24 ~ 0.64)	<0.001	0.39 (0.24 ~ 0.63)	<0.001
Q1 (0.05–0.46)	1 (Ref)		1 (Ref)		1 (Ref)		1 (Ref)		1 (Ref)		1 (Ref)	
Q2 (0.47–0.61)	0.63 (0.47 ~ 0.84)	0.001	0.63 (0.47 ~ 0.84)	0.002	0.64 (0.48 ~ 0.86)	0.003	0.65 (0.48 ~ 0.87)	0.004	0.61 (0.45 ~ 0.83)	0.002	0.63 (0.46 ~ 0.86)	0.004
Q3 (0.62–0.78)	0.6 (0.45 ~ 0.81)	0.001	0.63 (0.47 ~ 0.85)	0.002	0.7 (0.52 ~ 0.95)	0.021	0.7 (0.52 ~ 0.95)	0.024	0.63 (0.45 ~ 0.86)	0.005	0.64 (0.46 ~ 0.89)	0.008
Q4 (0.79–3.46)	0.49 (0.36 ~ 0.67)	<0.001	0.53 (0.39 ~ 0.73)	<0.001	0.58 (0.42 ~ 0.79)	0.001	0.5 (0.37 ~ 0.69)	<0.001	0.45 (0.32 ~ 0.65)	<0.001	0.44 (0.31 ~ 0.64)	<0.001
*P* for Trend		<0.001		<0.001		0.001		<0.001		<0.001		<0.001

^a^Cr/CysC ratio was entered as a continuous variable per 1 unit increase.

Model 1: Not adjusted.

Model 2: Adjusted for age, sex, BMI, smoking and drinking.

Model 3: Adjusted for model 2 + infection, hypertension, Coronary heart, diabetes, arrhythmia, stroke, COPD, liver disease, cancer.

Model 4: Adjusted for model 3 + Mechanical Ventilation, Vasoactive drug use, Heart rate, MAP, PaO_2_/FiO_2_, Temperature, Respiratory rate.

Model 5: Adjusted for model 4 + CRP, PLT, PCT, TBIL, ALB, PT, APTT, WBC, LAC, BUN, Uric acid, β_2_-MG.

Model 6: Adjusted for model 5 + AKI, CRRT.

Q, quartile; Cr/CysC ratio, Creatinine/cystatin C ratio; HR, hazard ratio; CI, confidence interval; Ref, reference.

The area under the ROC curve (AUC) for the Cr/CysC ratio in predicting 28-day mortality was 0.592 (Supplementary Figure S2). Consistent with these findings, Tables 3, 4 quantified the incremental predictive utility using NRI and IDI values. Specifically, both the APACHE II + Cr/CysC ratio and SOFA + Cr/CysC ratio models significantly improved risk reclassification: for APACHE II, NRI = 0.114 (95% CI: 0.024–0.177) and IDI = 0.012 (95% CI: 0.002–0.028); for SOFA, NRI = 0.008 (95% CI: 0.003–0.028) and IDI = 0.010 (95% CI: 0.001–0.027) (all *p* < 0.05).

**Table 3:** Incremental predictive value of APACHE II and Cr/CysC ratio + APACHE II for 28-day mortality with sepsis.

Model	IDI (95%CI)	*p* value	NRI (95%CI)	*p* value
APACHE II	Ref	Ref	Ref	Ref
Cr/CysC ratio + APACHE II	0.012 (0.002–0.028)	0.004	0.114 (0.024–0.177)	0.028

NRI, net reclassification improvement; Ref, reference; IDI, integrated discrimination improvement; CI, confidence interval; Ref, reference; Cr/CysC ratio, creatinine/cystatin C ratio.

**Table 4:** Incremental predictive value of SOFA and Cr/CysC ratio + SOFA for 28-day mortality with sepsis.

Model	IDI (95%CI)	*p* value	NRI (95%CI)	*p* value
SOFA	Ref	Ref	Ref	Ref
Cr/CysC ratio + SOFA	0.01 (0.001–0.027)	0.012	0.013 (0.003–0.028)	0.008

NRI, net reclassification improvement; Ref, reference; IDI, integrated discrimination improvement; CI, confidence interval; Ref, reference; Cr/CysC ratio, creatinine/cystatin C ratio.

**Figure 2:**
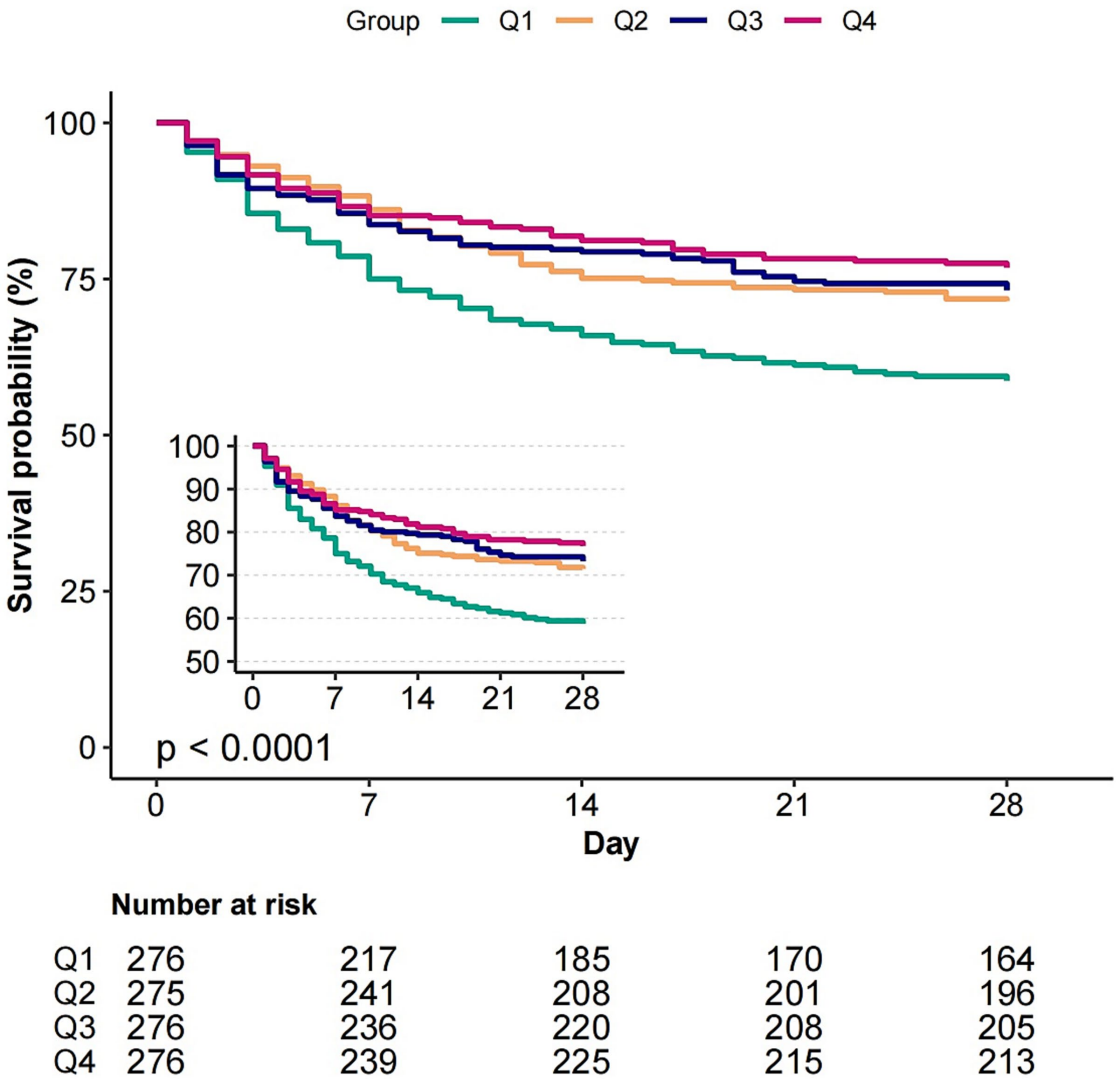
Kaplan–Meier survival analysis curves for 28-day mortality stratified by Cr/CysC ratio categories. Cr/CysC ratio (Q1: 0.05–0.46, Q2: 0.47–0.61, Q3: 0.62–0.78, and Q4: 0.79–3.46).

**Figure 3:**
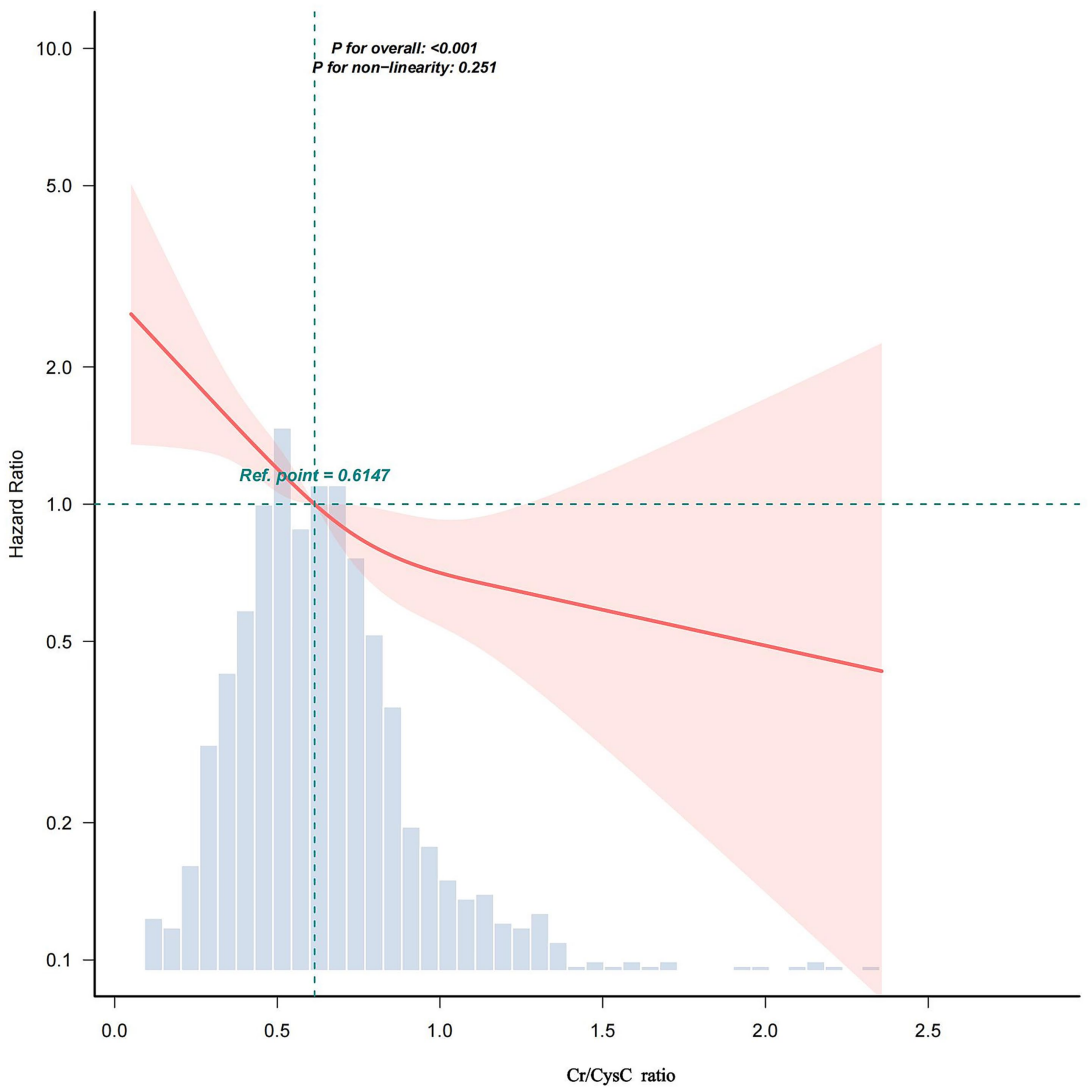
Association between the Cr/CysC ratio and 28-day mortality in critically ill patients with sepsis. Solid and dashed lines represent the predicted values and 95% confidence intervals, respectively. Adjusted for age, sex, BMI, smoking, drinking, infection, hypertension, coronary heart disease, diabetes, arrhythmia, stroke, COPD, liver disease, cancer, mechanical ventilation, vasoactive drug use, heart rate, MAP, PaO_2_/FiO_2_, temperature, Lac, PLT, PCT, TBIL, ALB, PT, APTT, CRP, WBC, BUN, uric acid, β_2_-MG, AKI, and CRRT.

In the univariate analysis, the Cr/CysC ratio was observed to be strongly associated with the 28-day risk of mortality, as illustrated by the Kaplan–Meier curves (log-rank test, *p* < 0.001; Figure 2). Spline analysis indicated an approximately linear inverse association between Cr/CysC ratio and 28-day mortality (*P* nonlinearity = 0.251) (Figure 3).

### 3.3 Subgroup analysis

**Figure 4:**
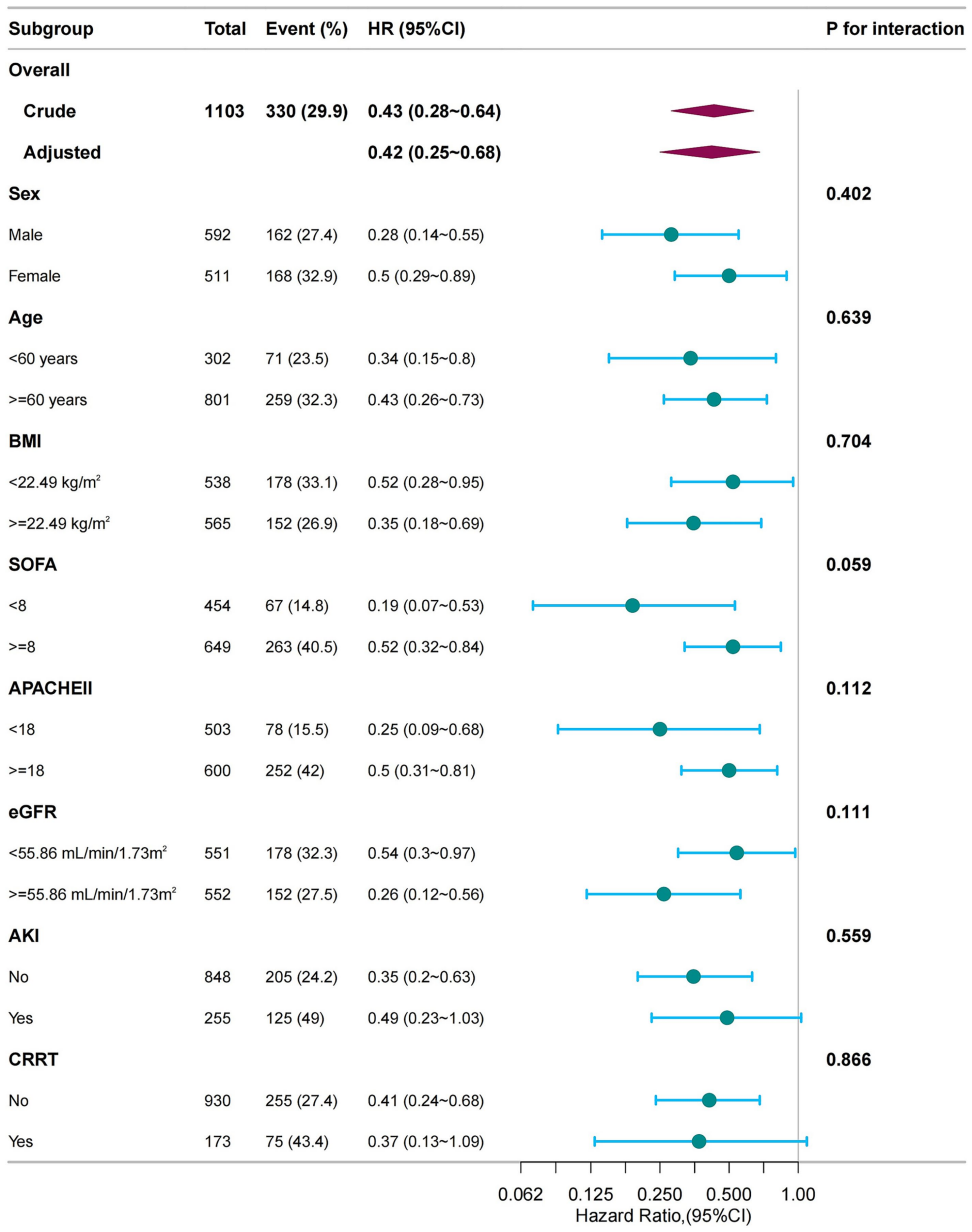
Subgroup analysis of the association between Cr/CysC ratio and 28-day mortality.

Subgroup analyses were performed to evaluate potential effect modification of the association between the Cr/CysC ratio and 28-day mortality. No significant interactions were observed in any subgroup after stratification by age, sex, BMI, admission year, APACHE II score, SOFA score, AKI, CRRT and eGFR (Figure 4).

### 3.4 Sensitivity analysis

Owing to the retrospective design, we excluded patients with missing creatinine or cystatin C measurements and those with less than 28 days’ follow-up. Baseline characteristics of included and excluded patients were broadly comparable (Supplementary Table S5). No significant differences were observed in demographics, comorbidities, vital signs, illness severity, organ support, or 28-day mortality (all *p* > 0.05), although statistically significant differences were noted in infection source, oxygenation (PaO_2_//FiO_2_), length of ICU stay, and several biomarkers (LAC, PLT, PCT, APTT, β_2_-MG; all *p* < 0.05).

As the study period (2017–2023) spanned the COVID-19 pandemic, we performed a sensitivity analysis by stratifying the cohort into two intervals (2017–2019 and 2020–2023) to account for potential temporal effects. Subgroup analyses revealed no significant interactions, supporting the robustness of our findings (Figure 3). Descriptive comparisons between periods showed significant differences in MAP, PCT, CRP, AKI incidence and CRRT use, whereas Cr/CysC and 28-day mortality did not differ significantly (Supplementary Table S6). The E-value for this point estimate is 4.85 and for the upper confidence interval limit is 2.61 (Supplementary Figure S1). Following VanderWeele (35), these values suggest that the association between the Cr/CysC ratio and 28-day mortality is unlikely to be fully explained by unmeasured confounders unless such confounders are strongly associated with both the exposure and outcome, with hazard ratios of at least 4.85, respectively.

To confirm that the correlation between the Cr/CysC ratio and survival was valid regardless of renal function at ICU admission, we reassessed this relationship in patients with eGFRs >60 mL/min/1.73 m^2^. The HR value per 1 unit increase in the Cr/CysC ratio for 28-day mortality was 0.2 (95% CI: 0.1–0.37). The HR value for 28-day mortality was observed to be sequentially lower in quartiles with higher Cr/CysC ratios. Moreover, for Q4, the HR value for 28-day mortality was 0.39 (95% CI: 0.27–0.58) compared with that for Q1. These correlations remained unchanged after adjusting for confounders (Supplementary Table S7). The Kaplan–Meier curves revealed that the 28-day survival probabilities were significantly lower for patients in Q1 than for those in the other quartiles (log-rank test, *p* < 0.001) (Supplementary Figure S3). The results were also stable in the subgroup analyses (Supplementary Figure S6). We included subgroups of patients according to the occurrence of AKI and performed a Cox multifactorial analysis (Supplementary Table S8). We observed similar associations between the Cr/CysC ratio and 28-day mortality in both subgroups and sensitivity analyses (Supplementary Figures S4, S5, S7, S8).

## 4 Discussion

In this study, the Cr/CysC ratio measured upon admission to the ICU was associated with 28-day mortality in patients with sepsis. Progressively higher Cr/CysC ratio were associated with decreasing hazard ratios for 28-day mortality. Compared with the lowest quartile, the risk of 28-day mortality was lower in the middle and highest quartiles of the Cr/CysC ratio; this association remained significant even after adjusting for potential confounding factors.

Previous studies have demonstrated that the Cr/CysC ratio was a biomarker for estimating muscle mass and can be used as a screening tool and prognostic indicator of skeletal muscle reduction (16, 36–38). Oh et al. (9) conducted a retrospective study and reported that skeletal muscle hypoplasia was associated with both short-term and long-term mortality in patients with infectious shock. A prospective cohort study by Cox et al. (39) examined the effect of sarcopenia on one-year mortality in critically ill patients with intra-abdominal sepsis, with 47 patients with sepsis being enrolled. Barreto et al. (40) showed that the sarcopenia index (SI) [(serum creatinine/serum cystatin C) *100] was a cost-effective, objective marker of malnutrition that can be used to assess nutritional risk in ICU patients, including those with sepsis. However, the underlying mechanisms and clinical relevance require further investigation in future research.

The relationship between the Cr/CysC ratio and mortality has been established in several other patient groups. A low Cr/CysC ratio is associated with higher mortality in cancer patients (41), adults with non-dialysis chronic kidney disease (42), and those with chronic obstructive pulmonary disease (23). Additionally, the Cr/CysC ratio has been shown to predict mortality in elderly female patients with septic shock (43). In the present study, after adjusting for all confounders, the Cr/CysC ratio remained associated with 28-day mortality in critically ill patients with sepsis, suggesting its potential as an exploratory prognostic indicator in this population. One possible explanation for this relationship is that the Cr/CysC ratio reflects muscle mass, which influences prognosis in various patient groups, particularly critically ill patients (44). Chun et al. (45) demonstrated that in patients with cholangitis-associated sepsis, low muscle mass was significantly associated with clinical outcomes, particularly regarding in-hospital mortality, thus supporting this hypothesis. However, in patients with sepsis in the intensive care unit, both serum creatinine and cystatin C concentrations are strongly influenced by renal function, acute kidney injury, exposure to continuous renal replacement therapy, hemodynamic instability, and catabolic state. Thus, the Cr/CysC ratio likely sits at the intersection of muscle mass, kidney function, and illness severity. Future prospective studies are needed to clarify the causal relationship between the Cr/CysC ratio and prognosis in critically ill patients with sepsis.

Serum creatinine and cystatin C levels are widely used as clinical laboratory measurements of renal function. When renal function is impaired, the renal clearance of creatinine and cystatin C decreases, thereby increasing their serum levels; this change may be clinically influential in patients with reduced renal function (27). However, patients with sepsis demonstrate a higher incidence of concomitant AKI (46, 47). Therefore, we performed subgroup analyses stratified by eGFR and AKI status and found that the relationship between the Cr/CysC ratio and mortality remained. Moreover, this correlation persisted even after adjusting for baseline renal parameters, suggesting that the association between the Cr/CysC ratio and prognosis may be independent of renal injury. However, these findings should be confirmed in subsequent large-scale, multicenter prospective studies.

This study has several limitations. First, as a single-center retrospective cohort study, it was unable to establish a causal relationship between the Cr/CysC ratio and survival. Second, the single-center retrospective design was inevitably subject to selection bias. Third, we collected laboratory data only within the first 24 h of ICU admission and did not dynamically monitor changes in creatinine and cystatin C levels, which may have limited our ability to capture their full prognostic impact. Finally, in critically ill patients with sepsis, both creatinine and cystatin C are substantially influenced by renal function, AKI, CRRT, hemodynamic instability, and catabolic state. Consequently, the Cr/CysC ratio likely reflects an interplay among muscle mass, renal biomarker handling, and overall disease severity rather than a single biological process. Although we observed an association between a lower Cr/CysC ratio and increased 28-day mortality, the underlying mechanisms remain unresolved. Large, prospective, multicenter studies incorporating direct measures of muscle mass and serial renal biomarker assessments are required to validate and clarify these findings.

In conclusion, in critically ill patients with sepsis, the Cr/CysC ratio on ICU admission was significantly associated with 28-day mortality. However, further validation in larger prospective studies is needed.

## Data Availability

The raw data supporting the conclusions of this article will be made available by the authors, without undue reservation.
