# Supplementary material for: Associations between the creatinine/cystatin C ratio and 28-day mortality in critically ill patients with sepsis: a retrospective cohort study
Source: Front Nutr. 2026 Jun 18;13:1699579. doi: 10.3389/fnut.2026.1699579 (PMC13322943; doi:10.3389/fnut.2026.1699579)
Supplement: Supplementary file 1 [file Table_1.doc]

**Supplementary Material**

**Associations between the** **creatinine/cystatin C ratio and 28-day mortality in critically ill patients with sepsis: a retrospective cohort study**

**Supplementary Table S1.** Details of missing data

**Supplementary Table S2.** The variance inflation factor (VIF) of variables

**Supplementary Table S3.** Baseline Characteristics of Included and Excluded Participants

**Supplementary Table S4.** Verification of Proportional Risk Assumptions

**Supplementary Table S5.** Results of univariate analysis of 28-day mortality

**Supplementary Table S6.** General Population Descriptions Across Different Time Periods

**Supplementary Table S7.** Cox proportional hazard regression analyses for 28-day mortality in eGFR subgroups

**Supplementary Table S8.** Cox proportional hazard regression analyses for 28-day mortality in sepsis patients with and without acute kidney injury

**Supplementary Figure S1.** The E‑values between the Cr/CysC ratio and 28‑day mortality

**Supplementary Figure S2.** The receiver operating characteristic (ROC) curves of the Cr/CysC ratio to predict 28-day mortality .

**Supplementary Figure S3.** COX regression analyses of 28-day mortality with adjustment for changes in eGFR ≥60mL/min/1.73m2.

**Supplementary Figure S4.** 28-day mortality as expressed in Cr/CysC ratio quintiles for sepsis patients without acute kidney injury

**Supplementary Figure S5.** 28-day mortality as expressed in Cr/CysC ratio quintiles for sepsis patients with acute kidney injury

**Supplementary Figure S6.** Stratified analyses of the association between Cr/CysC ratio and 28-day mortality status according to baseline characteristics in Sepsis database with eGFR ≥60mL/min/1.73m^2^.

**Supplementary Figure S7.** Stratified analyses of the association between Cr/CysC ratio and 28-day mortality status according to baseline characteristics in Sepsis database without acute kidney injury.

**Supplementary Figure S8.** Stratified analyses of the association between Cr/CysC ratio and 28-day mortality status according to baseline characteristics in Sepsis database with acute kidney injury.

**Supplementary Table S1. Details of missing data**

| Variable | Miss.freq | Miss.percentage% |
| --- | --- | --- |
| Age(years) | 0 | 0 |
| AKI(%) | 0 | 0 |
| ALB(g/L) | 0 | 0 |
| ALT(U/L) | 10 | 0.9066 |
| APACHE II | 0 | 0 |
| APTT(s） | 3 | 0.272 |
| BMII(Kg/m^2^) | 0 | 0 |
| Infection (%) | 1 | 0.0907 |
| Hypertension(%) | 0 | 0 |
| Coronary heart(%) | 2 | 0.1813 |
| Diabetes(%) | 1 | 0.0907 |
| Arrhythmia(%) | 2 | 0.1813 |
| Cancer(%) | 1 | 0.0907 |
| COPD(%)(%) | 0 | 0 |
| Stroke(%) | 1 | 0.0907 |
| Liver disease | 2 | 0.1813 |
| Creatinine(mg/L) | 0 | 0 |
| CRP(mg/L) | 5 | 0.4533 |
| CRRT(%) | 0 | 0 |
| Cystatin C(mg/L) | 0 | 0 |
| Drinking(%) | 0 | 0 |
| eGFRmL/min/1.73m^2^) | 0 | 0 |
| Heart rate(beats/min) | 0 | 0 |
| Hospital mortality(%) | 0 | 0 |
| Hospital Los(day) | 0 | 0 |
| ICU Los(day) | 0 | 0 |
| LAC (mmol/L) | 40 | 3.6265 |
| MAP(mmHg) | 0 | 0 |
| Mechanical Ventilation(%) | 0 | 0 |
| Vasoactive drug use(%) | 0 | 0 |
| PAO_2_/FIO_2_ | 0 | 0 |
| PCT(ng/ml) | 91 | 8.2502 |
| PLT (×10^9^ /L) | 0 | 0 |
| PT(s） | 3 | 0.272 |
| Sex(%) | 0 | 0 |
| Smoking(%) | 0 | 0 |
| SOFA | 0 | 0 |
| Temperature(◦C) | 0 | 0 |
| TBIL(umol/L) | 9 | 0.816 |
| BUN(mmol/L) | 9 | 0.816 |
| Uric acid(umol/L) | 24 | 2.1759 |
| 28-day mortality(%) | 0 | 0 |
| β_2_-MG(mg/L) | 25 | 2.2665 |
| WBC (×10^9^ /L) | 3 | 0.272 |
| Year | 0 | 0 |
| Respiratory rate(beats/min) | 0 | 0 |

BMI: body mass index ;SOFA: sequential organ failure assessment score;APACHE II,Acute Physiology and Chronic Health Score II;LAC:Lactic acid; MAP :mean blood pressure;COPD: chronic obstructive pulmonary disease; WBC: white blood cell; PLT:platelets; PCT:procalcitonin;ALT:alanine aminotransferase ;ALB:albumin;

TBIL:total bilirubin;PT :prothrombin time;APTT: activated partial thromboplastin time;BUN: blood urea nitrogen;AKI:acute kidney injury;β2-MG:β2-microglobulin

**Supplementary Table S2. The variance inflation factor (VIF) of variables**

| Variables | Variance inflation factor (VIF) |
| --- | --- |
| Age(years) | 1.422 |
| Sex (%) | 1.111 |
| BMI(Kg/m^2^) | 1.133 |
| Smoking (%) | 2.243 |
| Drinking(%) | 2.197 |
| Infection (%) | 1.526 |
| Heart rate(beats/min) | 1.323 |
| MAP (mmHg) | 1.178 |
| Respiratory rate(beats/min) | 1.311 |
| Temperature(◦C) | 1.091 |
| PAO_2_/FIO_2_ | 1.124 |
| Hypertension(%) | 1.364 |
| Coronary heart(%) | 2.961 |
| Diabetes(%) | 1.22 |
| Arrhythmian (%) | 2.762 |
| Stroke(%) | 1.302 |
| COPD(%) | 1.205 |
| Liver disease(%) | 1.16 |
| Cancer(%) | 1.162 |
| Mechanical Ventilation(%) | 1.48 |
| Vasoactive drug use(%) | 1.35 |
| LAC (mmol/L) | 1.465 |
| PLT (×10^9^ /L) | 1.246 |
| PCT(ng/ml) | 1.386 |
| ALB(g/L) | 1.226 |
| PT(s） | 2.162 |
| APTT(s） | 2.015 |
| TBIL(umol/L) | 1.319 |
| WBC (×10^9^ /L) | 1.143 |
| ALT(U/L) | 1.302 |
| CRP(mg/L) | 1.267 |
| BUN(mmol/L) | 1.649 |
| Uric acid(umol/L) | 1.514 |
| β_2_-MG(mg/L) | 1.356 |
| AKI (%) | 2.297 |
| CRRT(%) | 2.244 |

BMI: body mass index ;SOFA: sequential organ failure assessment score;APACHE II,Acute Physiology and Chronic Health Score II;LAC:Lactic acid; MAP :mean blood pressure;COPD: chronic obstructive pulmonary disease; WBC: white blood cell; PLT:platelets; PCT:procalcitonin;ALT:alanine aminotransferase ;ALB:albumin;

TBIL:total bilirubin;PT :prothrombin time;APTT: activated partial thromboplastin time;BUN: blood urea nitrogen;AKI:acute kidney injury;β2-MG:β2-microglobulin

**Supplementary Table S3. Baseline Characteristics of Included and Excluded Participants**

| Variables | Total  (n = 1372) | Excluded  (n = 269) | Included  (n = 1103) | p |
| --- | --- | --- | --- | --- |
| Age(years) | 66.8 ± 15.9 | 65.2 ± 15.2 | 67.1 ± 16.0 | 0.075 |
| Male (%) | 654 (47.7) | 141 (52.4) | 513 (46.5) | 0.082 |
| BMI(Kg/m^2^) | 22.8 ± 4.3 | 23.0 ± 4.3 | 22.8 ± 4.4 | 0.503 |
| Smoking (%) | 493 (36.0) | 87 (32.5) | 406 (36.8) | 0.18 |
| Drinking(%) | 470 (34.3) | 101 (37.5) | 369 (33.5) | 0.208 |
| **Infection (%)** |  |  |  | 0.006 |
| Blood | 73 ( 5.3) | 11 (4.1) | 62 (5.6) |  |
| Lung | 640 (46.7) | 107 (39.9) | 533 (48.4) |  |
| Abdominal | 410 (29.9) | 103 (38.4) | 307 (27.9) |  |
| Urinary tract | 165 (12.0) | 27 (10.1) | 138 (12.5) |  |
| Skin or soft tissue | 82 ( 6.0) | 20 (7.5) | 62 (5.6) |  |
| Heart rate(beats/min) | 107.7 ± 24.5 | 109.5 ± 20.7 | 107.3 ± 25.3 | 0.181 |
| MAP (mmHg) | 81.4 ± 20.8 | 73.4 ± 17.9 | 83.3 ± 20.9 | < 0.001 |
| Respiratory rate(breaths/min) | 24.3 ± 7.3 | 24.2 ± 7.4 | 24.3 ± 7.3 | 0.984 |
| Temperature(◦C) | 37.4 ± 3.8 | 37.5 ± 1.2 | 37.3 ± 4.7 | 0.654 |
| PAO_2_/FIO_2_ | 230.0 (170.0, 330.0) | 211.5 (166.0, 290.0) | 234.0 (174.0, 340.0) | 0.021 |
| Hypertension | 561 (40.9) | 103 (38.3) | 458 (41.5) | 0.333 |
| Coronary heart | 426 (31.1) | 78 (29) | 348 (31.6) | 0.14 |
| Diabetes | 391 (28.5) | 67 (24.9) | 324 (29.4) | 0.141 |
| Arrhythmian | 365 (26.6) | 68 (25.3) | 297 (27) | 0.578 |
| Stroke | 115 ( 8.4) | 15 (5.6) | 100 (9.1) | 0.064 |
| COPD | 139 (10.1) | 24 (8.9) | 115 (10.4) | 0.464 |
| Liver disease | 134 ( 9.8) | 21 (7.8) | 113 (10.3) | 0.226 |
| Mechanical Ventilatio(%) | 787 (57.7) | 158 (59.6) | 629 (57.2) | 0.47 |
| Vasoactive drug use(%) | 1011 (74.7) | 211 (79) | 800 (73.7) | 0.071 |
| APACHEII | 19.9 ± 8.1 | 20.4 ± 6.9 | 19.8 ± 8.3 | 0.316 |
| SOFA | 8.4 ± 3.8 | 8.4 ± 4.0 | 8.4 ± 3.7 | 0.897 |
| AKI, n (%) | 357 (27.0) | 78 (29.4) | 279 (26.4) | 0.328 |
| ICU Los(day) | 6.0 (3.0, 12.0) | 5.0 (2.0, 11.0) | 6.0 (3.0, 12.2) | 0.001 |
| Hospital Los(day) | 20.3 ± 55.9 | 15.5 ± 31.9 | 21.5 ± 60.3 | 0.112 |
| 28-day mortality (%) | 523 (40.4) | 88 (45.6) | 435 (39.4) | 0.108 |
| LAC (mmol/L) | 2.3 (1.4, 4.0) | 2.8 (2.1, 4.0) | 2.1 (1.3, 4.1) | < 0.001 |
| PLT (×10^9^ /L) | 158.0 (86.0, 240.2) | 146.0 (76.5, 218.0) | 161.0 (89.0, 247.0) | 0.014 |
| PCT(ng/ml) | 5.6 (0.7, 26.6) | 12.7 (1.5, 26.1) | 4.8 (0.6, 26.9) | 0.04 |
| ALB(g/L) | 29.2 (25.3, 33.2) | 28.9 (25.3, 32.6) | 29.3 (25.3, 33.3) | 0.253 |
| PT(s） | 14.5 (13.0, 16.8) | 14.5 (12.8, 17.3) | 14.5 (13.0, 16.6) | 0.863 |
| APTT(s） | 35.2 (30.1, 42.1) | 38.6 (31.3, 48.4) | 34.8 (30.0, 40.7) | < 0.001 |
| TBIL(umol/L) | 17.0 (10.6, 32.5) | 17.3 (11.3, 30.8) | 17.0 (10.5, 32.8) | 0.809 |
| WBC (×10^9^ /L) | 11.4 (6.8, 17.1) | 12.2 (6.6, 19.5) | 11.3 (6.9, 16.8) | 0.206 |
| ALT(U/L) | 29.0 (16.1, 66.7) | 33.5 (18.5, 81.5) | 28.0 (15.9, 62.2) | 0.045 |
| CRP(mg/L) | 106.2 (38.0, 177.5) | 107.0 (37.0, 180.0) | 106.0 (38.2, 176.6) | 0.968 |
| BUN(mmol/L) | 9.3 (6.2, 13.9) | 10.1 (7.2, 13.6) | 9.1 (5.9, 13.9) | 0.106 |
| Creatinine(mg/L) | 85.5 (59.0, 122.0) | 87.9 (67.7, 120.5) | 84.5 (57.3, 123.4) | 0.127 |
| Cystatin C(mg/L) | 1.6 (1.2, 2.1) | 2.0 (1.4, 2.1) | 1.6 (1.2, 2.1) | 0.193 |
| Uric acid(umol/L) | 273.0 (188.0, 375.8) | 297.5 (190.0, 410.8) | 271.0 (187.8, 369.0) | 0.146 |
| β_2_-MG(mg/L) | 3.8 (2.6, 6.4) | 6.0 (3.6, 8.3) | 3.7 (2.5, 6.2) | < 0.001 |

BMI: body mass index ;SOFA: sequential organ failure assessment score;APACHE II,Acute Physiology and Chronic Health Score II;LAC:Lactic acid; MAP :mean blood pressure;COPD: chronic obstructive pulmonary disease; WBC: white blood cell; PLT:platelets; PCT:procalcitonin;ALT:alanine aminotransferase ;ALB:albumin;

TBIL:total bilirubin;PT :prothrombin time;APTT: activated partial thromboplastin time;BUN: blood urea nitrogen;AKI:acute kidney injury;β_2_-MG:β_2_-microglobulin ;Cr/CysC ratio:Creatinine/cystatin C ratio

**Supplementary Table S4. Verification of Proportional Risk Assumptions**

| Variable | chisq | df | *P* value |
| --- | --- | --- | --- |
| Cr/CysC ratio | 3.493 | 1 | 0.062 |
| GLOBAL | 3.493 | 1 | 0.062 |

**Supplementary Table S5. Results of univariate analysis of 28-day mortality**

| **Characteristic** | **HR 95% CI** | ***P-value*** |
| --- | --- | --- |
| Age(years) | 1.0089 (1.0016,1.0162) | 0.016 |
| Male | 1.23 (0.99,1.53) | 0.056 |
| BMI(Kg/m^2^) | 0.95 (0.92,0.97) | < 0.001 |
| Smoking | 1.15 (0.92,1.44) | 0.218 |
| Drinking | 1.16 (0.92,1.45) | 0.212 |
| Infection: ref.= Blood |  |  |
| Lung | 2.89 (1.36,6.15) | 0.006 |
| Abdominal | 1.39 (0.64,3.02) | 0.408 |
| Urinary tract | 1.17 (0.51,2.69) | 0.716 |
| Skin or soft tissue | 2.47 (1.08,5.65) | 0.033 |
| Hypertension | 0.9951 (0.7969,1.2426) | 0.966 |
| Coronary heart | 1.15 (0.91,1.45) | 0.255 |
| Diabetes | 0.86 (0.66,1.1) | 0.227 |
| Arrhythmian | 1.09 (0.86,1.39) | 0.468 |
| Stroke | 1.21 (0.96,1.54) | 0.111 |
| COPD | 1.28 (0.91,1.81) | 0.158 |
| Liver disease | 1.31 (0.95,1.8) | 0.101 |
| Cancer | 1.28 (0.91,1.8) | 0.15 |
| Heart rate(beats/min) | 1.01 (1.01,1.02) | < 0.001 |
| MAP (mmHg) | 1.0019 (0.9964,1.0074) | 0.501 |
| Temperature(◦C) | 1.01 (0.99,1.03) | 0.346 |
| PAO_2_/FIO_2_ | 0.9964 (0.9953,0.9974) | < 0.001 |
| Resp rate(beats/min) | 1.03 (1.02,1.05) | < 0.001 |
| Mechanical Ventilation | 3.09 (2.38,4) | < 0.001 |
| Vasoactive drug use | 2.07 (1.59,2.69) | < 0.001 |
| APACHEII | 1.07 (1.06,1.08) | < 0.001 |
| SOFA | 1.19 (1.16,1.23) | < 0.001 |
| LAC (mmol/L) | 1.12 (1.1,1.15) | < 0.001 |
| PCT(ng/ml) | 0.9982 (0.9948,1.0016) | 0.295 |
| PLT (×10^9^ /L) | 0.9984 (0.9974,0.9993) | < 0.001 |
| CRP(mg/L) | 1.0007 (0.9994,1.002) | 0.317 |
| WBC (×10^9^ /L) | 1.0057 (0.9935,1.0181) | 0.362 |
| PT(s） | 1.04 (1.03,1.06) | < 0.001 |
| APTT(s） | 1.03 (1.02,1.04) | < 0.001 |
| ALB(g/L) | 0.98 (0.96,1) | 0.014 |
| TBIL(umol/L) | 1.0025 (1.0005,1.0045) | 0.012 |
| Creatinine(mg/L) | 1.25 (1.00,1.55) | 0.047 |
| Cystatin C(mg/L) | 1.26 (1.16,1.37) | < 0.001 |
| BUN(mmol/L) | 1.03 (1.01,1.04) | < 0.001 |
| Uric acid(umol/L) | 1.0016 (1.0008,1.0023) | < 0.001 |
| β_2_-MG(mg/L) | 1.02 (1.01,1.03) | < 0.001 |
| Cr/CysC ratio | 0.42 (0.27,0.64) | < 0.001 |
| eGFR(mL/min/1.73m^2^) | 1.0003 (0.9991,1.0016) | 0.595 |
| AKI | 2.4 (1.92,2.99) | < 0.001 |
| CRRT | 1.77 (1.37,2.29) | < 0.001 |

BMI: body mass index ;SOFA: sequential organ failure assessment score;APACHE II,Acute Physiology and Chronic Health Score II;LAC:Lactic acid; MAP :mean blood pressure;COPD: chronic obstructive pulmonary disease; WBC: white blood cell; PLT:platelets; PCT:procalcitonin;ALT:alanine aminotransferase ;ALB:albumin;

TBIL:total bilirubin;PT :prothrombin time;APTT: activated partial thromboplastin time;BUN: blood urea nitrogen;AKI:acute kidney injury;β_2_-MG:β_2_-microglobulin ;Cr/CysC ratio:Creatinine/cystatin C ratio

**Supplementary Table S6. General Population Descriptions Across Different Time Periods**

| Variables | Total  (n = 1103) | 2017-2019  (n = 267) | 2020-2023  (n = 836) | *p* |
| --- | --- | --- | --- | --- |
| Age(years) | 67.1 ± 15.8 | 66.4 ± 15.5 | 67.3 ± 15.9 | 0.409 |
| Male (%) | 511 (46.3) | 132 (49.4) | 379 (45.3) | 0.242 |
| BMI(Kg/m^2^) | 22.7 ± 4.4 | 22.8 ± 4.1 | 22.7 ± 4.4 | 0.82 |
| Smoking (%) | 376 (34.1) | 84 (31.5) | 292 (34.9) | 0.298 |
| Drinking(%) | 355 (32.2) | 84 (31.5) | 271 (32.4) | 0.771 |
| **Infection (%)** |  |  |  | 0.245 |
| Blood | 44 ( 4.0) | 9 (3.4) | 35 (4.2) |  |
| Lung | 506 (45.9) | 109 (40.8) | 397 (47.5) |  |
| Abdominal | 329 (29.8) | 93 (34.8) | 236 (28.2) |  |
| Urinary tract | 144 (13.1) | 37 (13.9) | 107 (12.8) |  |
| Skin or soft tissue | 80 ( 7.3) | 19 (7.1) | 61 (7.3) |  |
| Heart rate(beats/min) | 107.3 ± 24.8 | 108.4 ± 24.1 | 107.0 ± 25.0 | 0.401 |
| MAP (mmHg) | 82.9 ± 19.8 | 79.5 ± 17.7 | 84.0 ± 20.3 | 0.001 |
| Respiratory rate(breaths/min) | 24.3 ± 7.3 | 23.8 ± 7.0 | 24.4 ± 7.4 | 0.242 |
| Temperature(◦C) | 37.5 ± 2.8 | 37.5 ± 1.1 | 37.5 ± 3.2 | 0.851 |
| PAO_2_/FIO_2_ | 233.0 (171.0, 340.0) | 231.0 (163.0, 335.0) | 233.0 (175.0, 340.2) | 0.572 |
| Hypertensionn | 418 (37.9) | 88 (33) | 330 (39.5) | 0.056 |
| Coronary heart | 299 (27.1) | 60 (22.5) | 239 (28.6) | 0.05 |
| Diabetes | 294 (26.7) | 69 (25.8) | 225 (26.9) | 0.73 |
| Arrhythmian | 296 (26.8) | 45 (16.9) | 251 (30) | < 0.001 |
| Stroke | 277 (25.1) | 61 (22.8) | 216 (25.8) | 0.327 |
| COPD | 100 ( 9.1) | 25 (9.4) | 75 (9) | 0.846 |
| Liver disease | 120 (10.9) | 35 (13.1) | 85 (10.2) | 0.179 |
| Cancer | 110 (10.0) | 27 (10.1) | 83 (9.9) | 0.93 |
| Mechanical Ventilatio(%) | 622 (56.4) | 156 (58.4) | 466 (55.7) | 0.441 |
| Vasoactive drug use (%) | 727 (65.9) | 189 (70.8) | 538 (64.4) | 0.054 |
| LAC (mmol/L) | 2.0 (1.3, 3.5) | 2.3 (1.4, 4.0) | 1.8 (1.3, 3.4) | 0.013 |
| PLT (×10^9^ /L) | 163.0 (89.5, 253.0) | 157.0 (89.5, 261.0) | 165.0 (89.8, 248.0) | 0.884 |
| PCT(ng/ml) | 5.3 (0.6, 29.8) | 8.0 (1.4, 39.5) | 4.2 (0.6, 27.5) | < 0.001 |
| ALB(g/L) | 29.1 (25.1, 33.1) | 28.3 (24.7, 32.1) | 29.4 (25.3, 33.4) | 0.022 |
| PT(s） | 14.5 (13.0, 16.4) | 14.3 (12.9, 16.2) | 14.5 (13.1, 16.6) | 0.181 |
| APTT(s） | 34.5 (29.9, 40.0) | 35.1 (29.8, 42.7) | 34.5 (29.9, 39.4) | 0.064 |
| TBIL(umol/L) | 17.0 (10.8, 30.4) | 17.6 (11.6, 30.1) | 16.9 (10.7, 30.4) | 0.46 |
| WBC (×10^9^ /L) | 11.2 (7.0, 16.6) | 11.8 (7.8, 17.6) | 11.1 (6.6, 16.3) | 0.047 |
| ALT(U/L) | 27.1 (15.4, 58.2) | 30.0 (16.5, 61.2) | 26.0 (15.1, 57.5) | 0.186 |
| CRP(mg/L) | 110.9 (39.5, 180.0) | 133.6 (62.9, 190.0) | 101.3 (35.5, 173.9) | < 0.001 |
| BUN(mmol/L) | 9.0 (5.8, 13.7) | 9.2 (5.5, 13.0) | 8.9 (5.9, 13.9) | 0.653 |
| Uric acid(umol/L) | 268.0 (189.5, 365.0) | 261.0 (182.0, 355.5) | 272.0 (190.8, 369.2) | 0.301 |
| β_2_-MG(mg/L) | 3.8 (2.6, 6.0) | 4.0 (2.7, 6.3) | 3.7 (2.6, 5.9) | 0.094 |
| Cr/CysC ratio | 0.6 (0.5, 0.8) | 0.6 (0.4, 0.8) | 0.6 (0.5, 0.8) | 0.89 |
| eGFR(mL/min/1.73m2) | 85.3 (55.5, 104.3) | 86.0 (53.6, 104.6) | 85.2 (55.7, 103.9) | 0.834 |
| APACHEII | 19.5 ± 8.1 | 19.3 ± 7.8 | 19.5 ± 8.2 | 0.781 |
| SOFA | 8.5 ± 3.6 | 8.7 ± 3.6 | 8.5 ± 3.6 | 0.339 |
| AKI, n (%) | 255 (23.1) | 84 (31.5) | 171 (20.5) | < 0.001 |
| CRRT, (%) | 173 (15.7) | 62 (23.2) | 111 (13.3) | < 0.001 |
| 28-day mortality (%) | 330 (29.9) | 70 (26.2) | 260 (31.1) | 0.129 |

BMI: body mass index ;SOFA: sequential organ failure assessment score;APACHE II,Acute Physiology and Chronic Health Score II;LAC:Lactic acid; MAP :mean blood pressure;COPD: chronic obstructive pulmonary disease; WBC: white blood cell; PLT:platelets; PCT:procalcitonin;ALT:alanine aminotransferase ;ALB:albumin;

TBIL:total bilirubin;PT :prothrombin time;APTT: activated partial thromboplastin time;BUN: blood urea nitrogen;AKI:acute kidney injury;β_2_-MG:β_2_-microglobulin ;Cr/CysC ratio:Creatinine/cystatin C ratio

**Supplementary Table S7. Cox proportional hazard regression analyses for 28-day mortality in eGFR subgroups**

| eGFR60≥  mL/min/1.73m^2^ | **Cr/CysC ratio** | |  | **Cr/CysC ratio quartiles** | | | | | | | |
| --- | --- | --- | --- | --- | --- | --- | --- | --- | --- | --- | --- |
|  | **HR(95%CI)** | ***p*** |  | **Reference** | **HR(95%CI)** | ***p*** | **HR(95%CI)** | ***p*** | **HR(95%CI)** | ***p*** | ***P* for**  **trend** |
|  | **(n=787)** | |  | **Q1(n=197)** | **Q2(n =196)** | | **Q3(n =197)** | | **Q4(n =197)** | |  |
| **Model 1** | 0.2(0.1~0.37) | <0.001 |  | **1(Ref)** | 0.67 (0.48~0.94) | 0.019 | 0.51 (0.36~0.74) | <0.001 | 0.39 (0.27~0.58) | <0.001 | <0.001 |
| **Model 2** | 0.22 (0.12~0.41) | **<0.001** |  | **1(Ref)** | 0.68 (0.49~0.96) | 0.027 | 0.54 (0.37~0.77) | 0.001 | 0.43 (0.29~0.63) | <0.001 | <0.001 |
| **Model 3** | 0.22 (0.12~0.45) | <0.001 |  | **1(Ref)** | 0.69(0.49~0.97) | 0.031 | 0.6 (0.42~0.87) | 0.007 | 0.465(0.3~0.67) | <0.001 | <0.001 |
| **Model 4** | 0.22 (0.12~0.42) | <0.001 |  | **1(Ref)** | 0.75 (0.53~1.08) | 0.12 | 0.64 (0.44~0.93) | 0.019 | 0.41 (0.27~0.62) | <0.001 | <0.001 |
| **Model 5** | 0.2(0.1~0.41) | <0.001 |  | **1(Ref)** | 0.82(0.56~1.18) | 0.278 | 0.64(0.43~0.95) | 0.029 | 0.4 (0.26~0.63) | <0.001 | <0.001 |

Model 1: Not adjusted.

Model 2: Adjusted for age, sex, BMI,smoking and drinking.

Model 3 :Adjusted for model 2 +infection,hypertension,Coronary heart,diabetes,arrhythmia,stroke,COPD,liver disease,cancer.

|  |
| --- |

Model 4 :Adjusted for model 3 + Mechanical Ventilation,Vasoactive drug use,heart rate,MAP,Pao2/Fio2,Temperature,RR.

Model 5:Adjusted for model 4+CRP, PLT,PCT,TBIL,ALB,PT,APTT,WBC,LAC,BUN,uric acid,β_2_-MG.

**Abbreviations:** Q: quartile; Cr/CysC ratio: Creatinine/cystatin C ratio; HR: hazard ratio; CI: confidence interval; Ref: reference

**Supplementary Table S8. Cox proportional hazard regression analyses for 28-day mortality in sepsis patients with and without acute kidney injury**

|  | **Cr/CysC ratio** | |  | **Cr/CysC ratio quartiles** | | | | | | | |
| --- | --- | --- | --- | --- | --- | --- | --- | --- | --- | --- | --- |
|  | **HR(95%CI)** | ***p*** |  | **Reference** | **HR(95%CI)** | ***p*** | **HR(95%CI)** | ***p*** | **HR(95%CI)** | ***p*** | ***P* for**  **trend** |
| **No AKI** | **(n=848)** | |  | **Q1(n=212)** | **Q2(n =212)** | | **Q3(n =212)** | | **Q4(n =212)** | |
| **Model 1** | 0.33 (0.18~0.58) | <0.001 |  | **1(Ref)** | 0.59 (0.41~0.85) | 0.005 | 0.58 (0.40~0.83) | 0.003 | 0.42 (0.28~0.62) | <0.001 | <0.001 |
| **Model 2** | 0.35 (0.19~0.62) | <0.001 |  | **1(Ref)** | 0.6 (0.42~0.86) | 0.005 | 0.58 (0.4~0.84) | 0.004 | 0.44(0.3~0.66) | <0.001 | <0.001 |
| **Model 3** | 0.42 (0.22~0.71) | 0.002 |  | **1(Ref)** | 0.6 (0.42~0.87) | 0.006 | 0.64 (0.44~0.94) | 0.024 | 0.48(0.31~0.72) | <0.001 | 0.001 |
| **Model 4** | 0.36(0.2~0.65) | 0.001 |  | **1(Ref)** | 0.59 (0.40~0.86) | 0.007 | 0.69 (0.47~1.01) | 0.059 | 0.45 (0.29~0.68) | <0.001 | 0.001 |
| **Model 5** | 0.32 (0.17~0.62) | 0.001 |  | **1(Ref)** | 0.55(0.37~0.82) | 0.004 | 0.64(0.43~0.96) | 0.033 | 0.41 (0.26~0.65) | <0.001 | <0.001 |
| **AKI** | **(n=225)** | |  | **Q1(n=64)** | **Q2(n =63)** | | **Q3(n =64)** | | **Q4(n =64)** | |  |
| **Model 1** | 0.43 (0.22~0.85) | 0.015 |  | **1(Ref)** | 0.54 (0.34~0.87) | 0.011 | 0.55 (0.34~0.88) | 0.013 | 0.39 (0.24~0.65) | <0.001 | <0.001 |
| **Model 2** | 0.49 (0.25~0.96) | 0.037 |  | **1(Ref)** | 0.57 (0.36~0.92) | 0.021 | 0.57 (0.35~0.92) | 0.021 | 0.45 (0.27~0.76) | 0.003 | 0.003 |
| **Model 3** | 0.57 (0.28~1.16) | 0.122 |  | **1(Ref)** | 0.52 (0.31~0.86) | 0.011 | 0.57 (0.34~0.94) | 0.028 | 0.48 (0.28~0.83) | 0.009 | 0.01 |
| **Model 4** | 0.37 (0.18~0.76) | 0.007 |  | **1(Ref)** | 0.62 (0.37~1.06) | 0.079 | 0.46 (0.28~0.77) | 0.003 | 0.37 (0.21~0.64) | <0.001 | <0.001 |
| **Model 5** | 0.49 (0.22~1.1) | 0.083 |  | **1(Ref)** | 0.6 (0.32~1.1) | 0.1 | 0.38(0.21~0.7) | 0.002 | 0.42 (0.22~0.8) | 0.009 | 0.002 |

Model 1: Not adjusted.

Model 2: Adjusted for age, sex, BMI,smoking and drinking.

Model 3 :Adjusted for model 2 +infection,hypertension,Coronary heart,diabetes,arrhythmia,stroke,COPD,liver disease,cancer.

|  |
| --- |

Model 4 :Adjusted for model 3 + Mechanical Ventilation,Vasoactive drug use,heart rate,MAP,Pao2/Fio2,Temperature,RR.

Model 5:Adjusted for model 4+CRP, PLT,PCT,TBIL,ALB,PT,APTT,WBC,LAC,BUN,uric acid,β_2_-MG.

**Abbreviations:** Q: quartile; Cr/CysC ratio: Creatinine/cystatin C ratio; HR: hazard ratio; CI: confidence interval; Ref: reference

|  |
| --- |

**
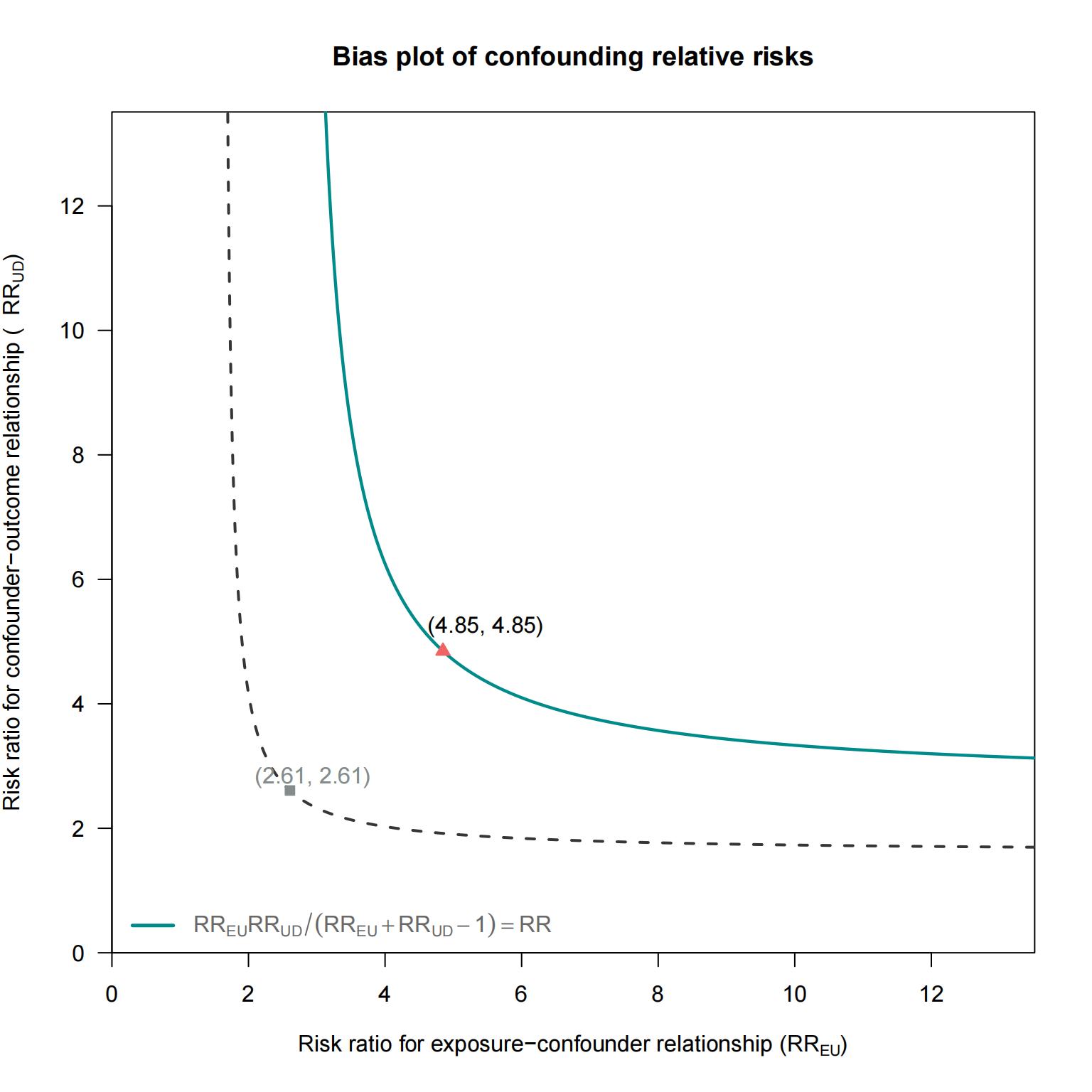
**

**Supplementary Figure S1. The E‑values between the Cr/CysC ratio and 28‑day mortality**


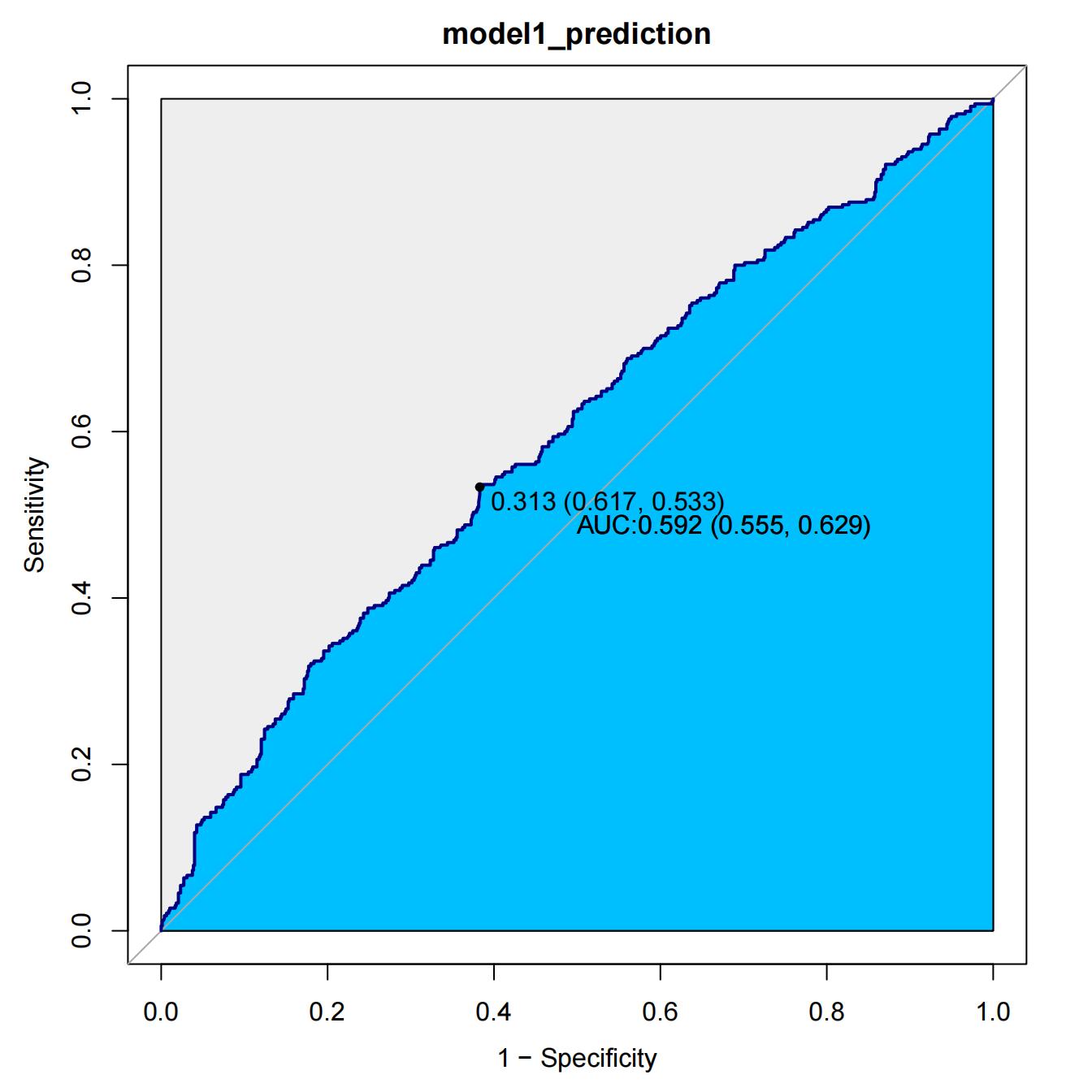


**Supplementary Figure S2. The receiver operating characteristic (ROC) curves of the Cr/CysC ratio to predict 28-day mortality .**


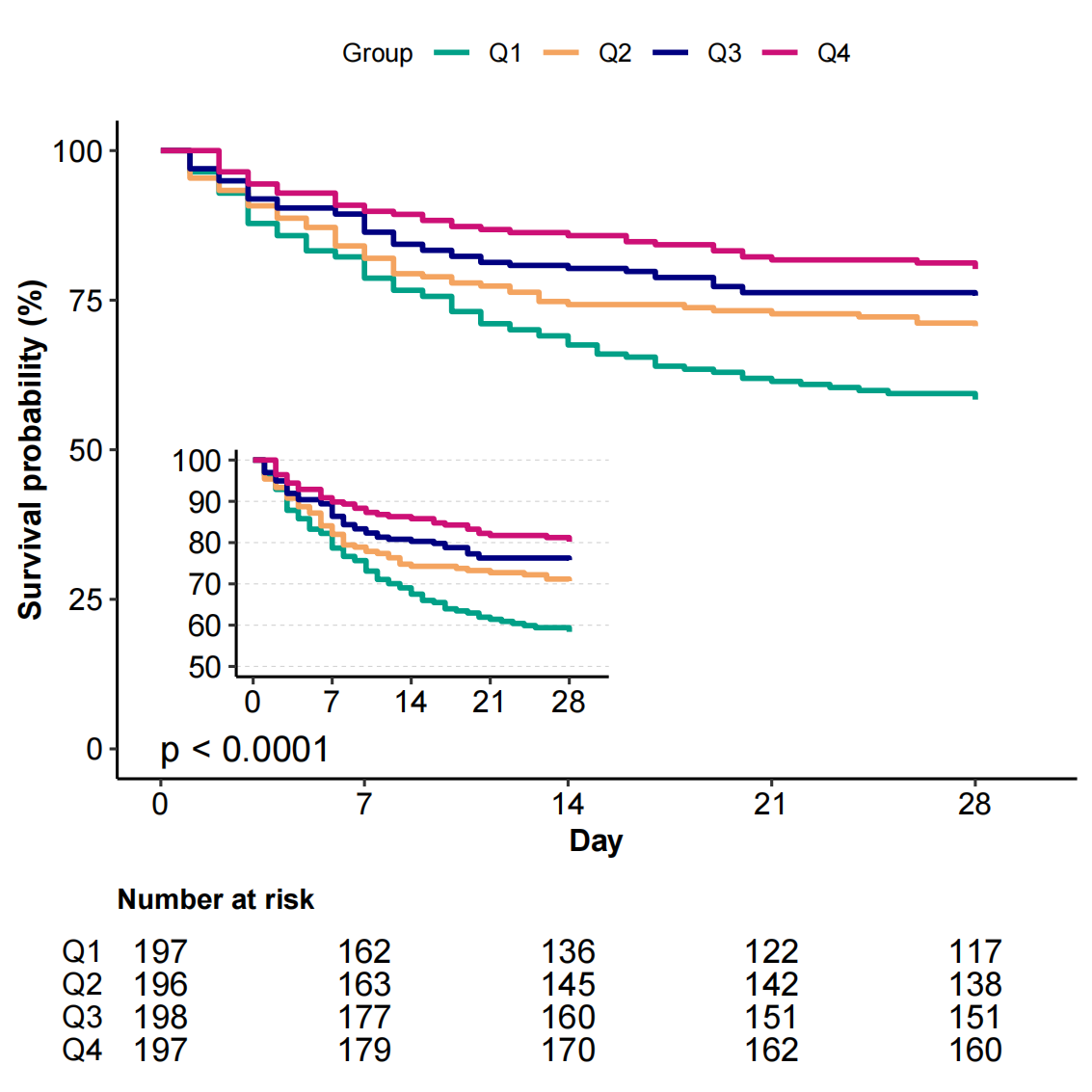


**Supplementary Figure S3. COX regression analyses of 28-day mortality with adjustment for changes in eGFR ≥60mL/min/1.73m^2^.**

**
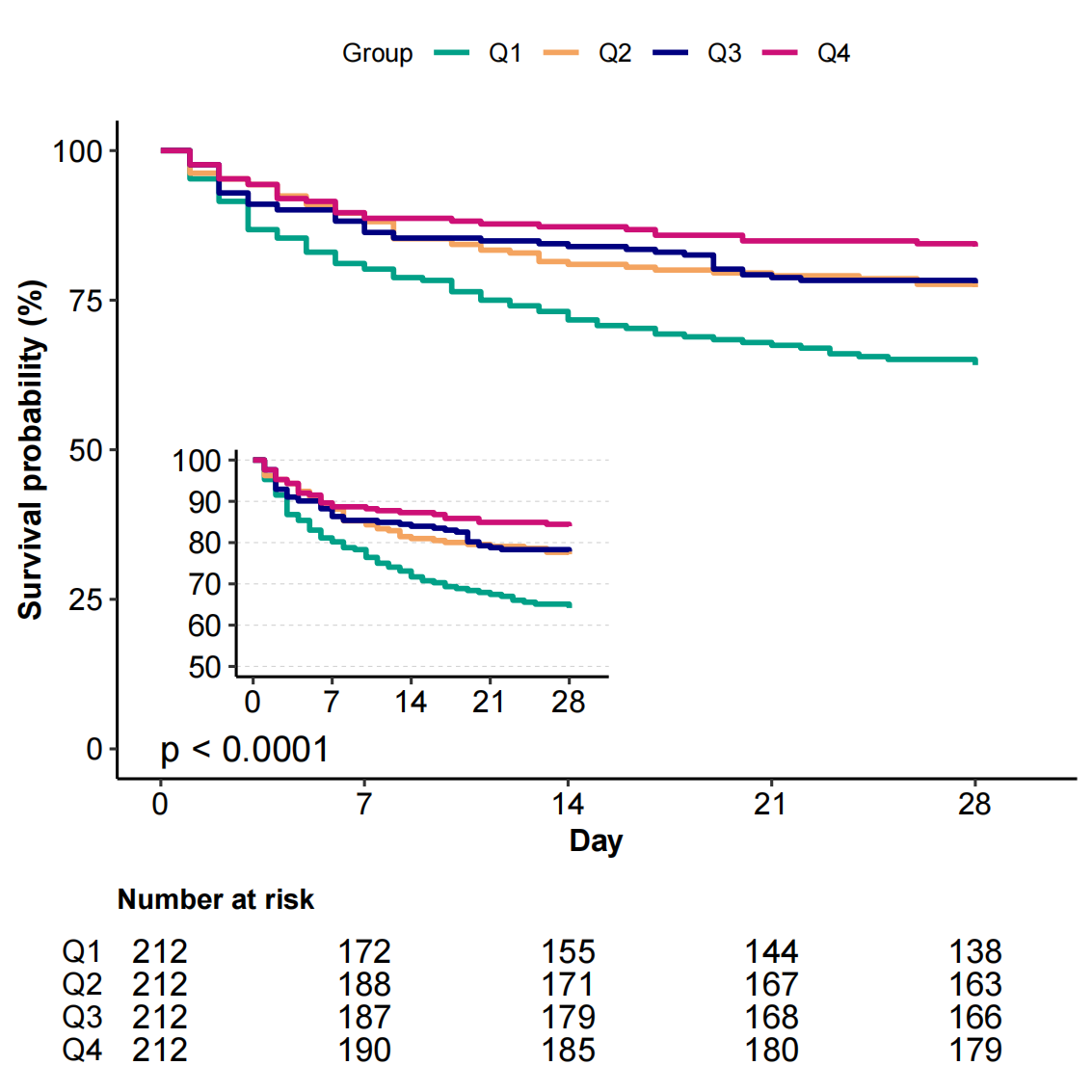
**

**Supplementary Figure S4. 28-day mortality as expressed in Cr/CysC ratio quintiles for sepsis patients without acute kidney injury**


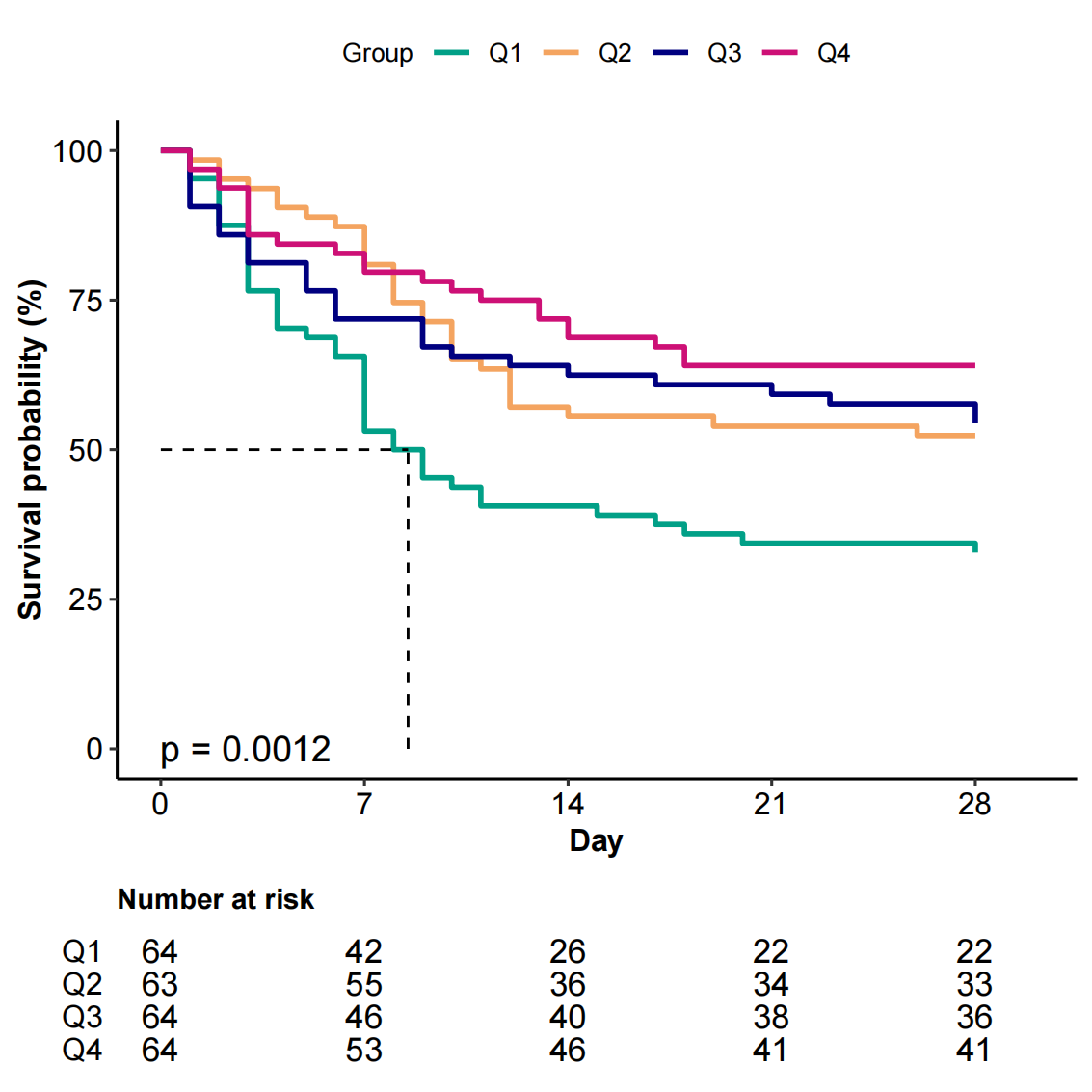


**Supplementary Figure S5. 28-day mortality as expressed in Cr/CysC ratio quintiles for sepsis patients with acute kidney injury**


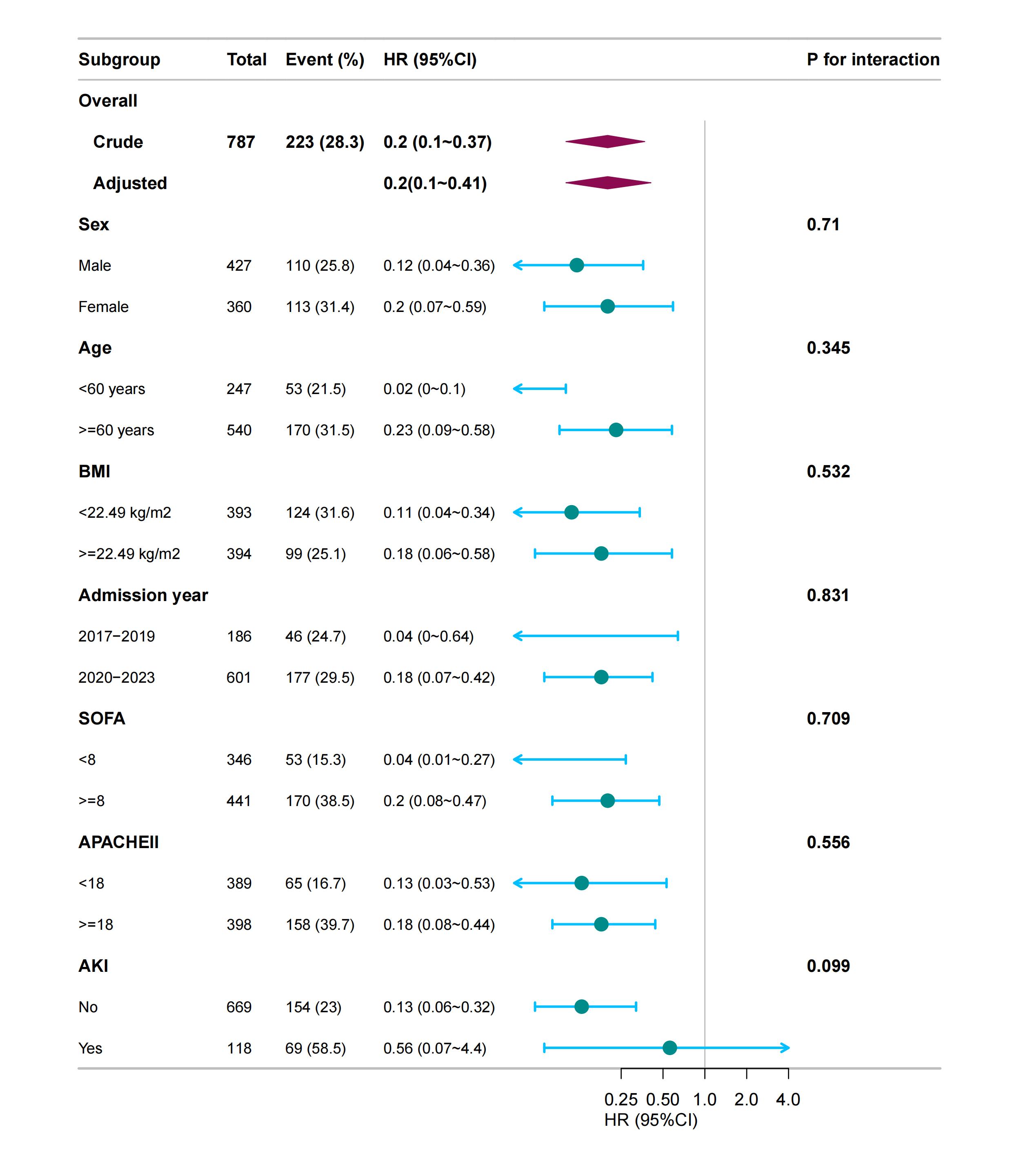


**Supplementary Figure S6. Stratified analyses of the association between Cr/CysC ratio and 28-day mortality status according to baseline characteristics in Sepsis database with eGFR ≥60mL/min/1.73m^2^.**

Adjusted for age, sex, BMI,smoking ,drinking, infection,hypertension,Coronary heart,diabetes,

arrhythmia,stroke,COPD,liver disease,cancer,Mechanical Ventilation,Vasoactive drug use,heart rate,MAP,Pao_2_/Fio_2_,Temperature,Lac,PLT,PCT,TBIL,ALB,PT,APTT,CRP,WBC, BUN,Uricacid,

β_2_-MG(model 5).

**
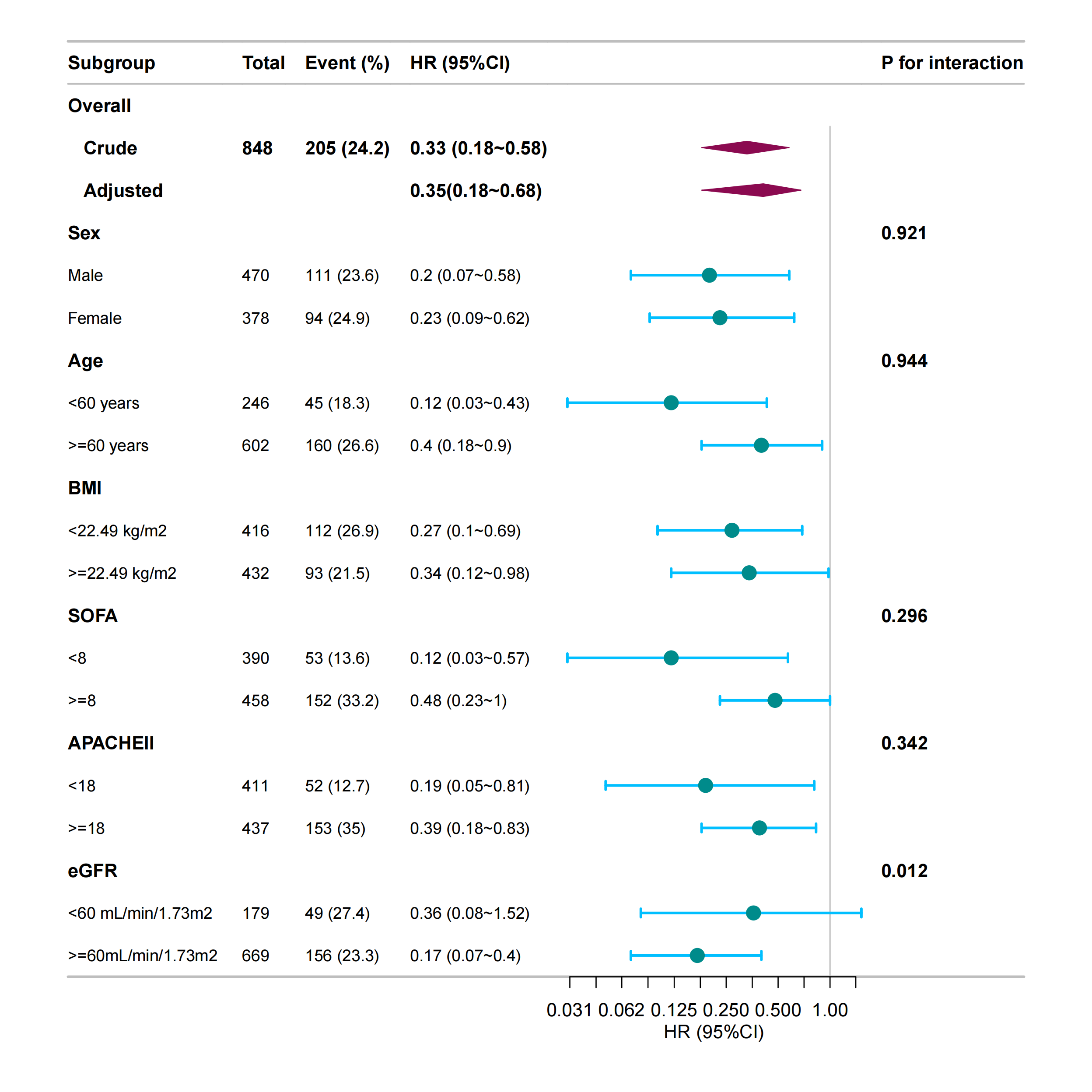
**

**Supplementary Figure S7. Stratified analyses of the association between Cr/CysC ratio and 28-day mortality status according to baseline characteristics in Sepsis database without acute kidney injury.**

Adjusted for age, sex, BMI,smoking ,drinking, infection,hypertension,Coronary heart,diabetes,

arrhythmia,stroke,COPD,liver disease,cancer,Mechanical Ventilation,Vasoactive drug use,heart rate,MAP,Pao_2_/Fio_2_,Temperature,Lac,PLT,PCT,TBIL,ALB,PT,APTT,CRP,WBC, BUN,Uricacid,

β_2_-MG(model 5).


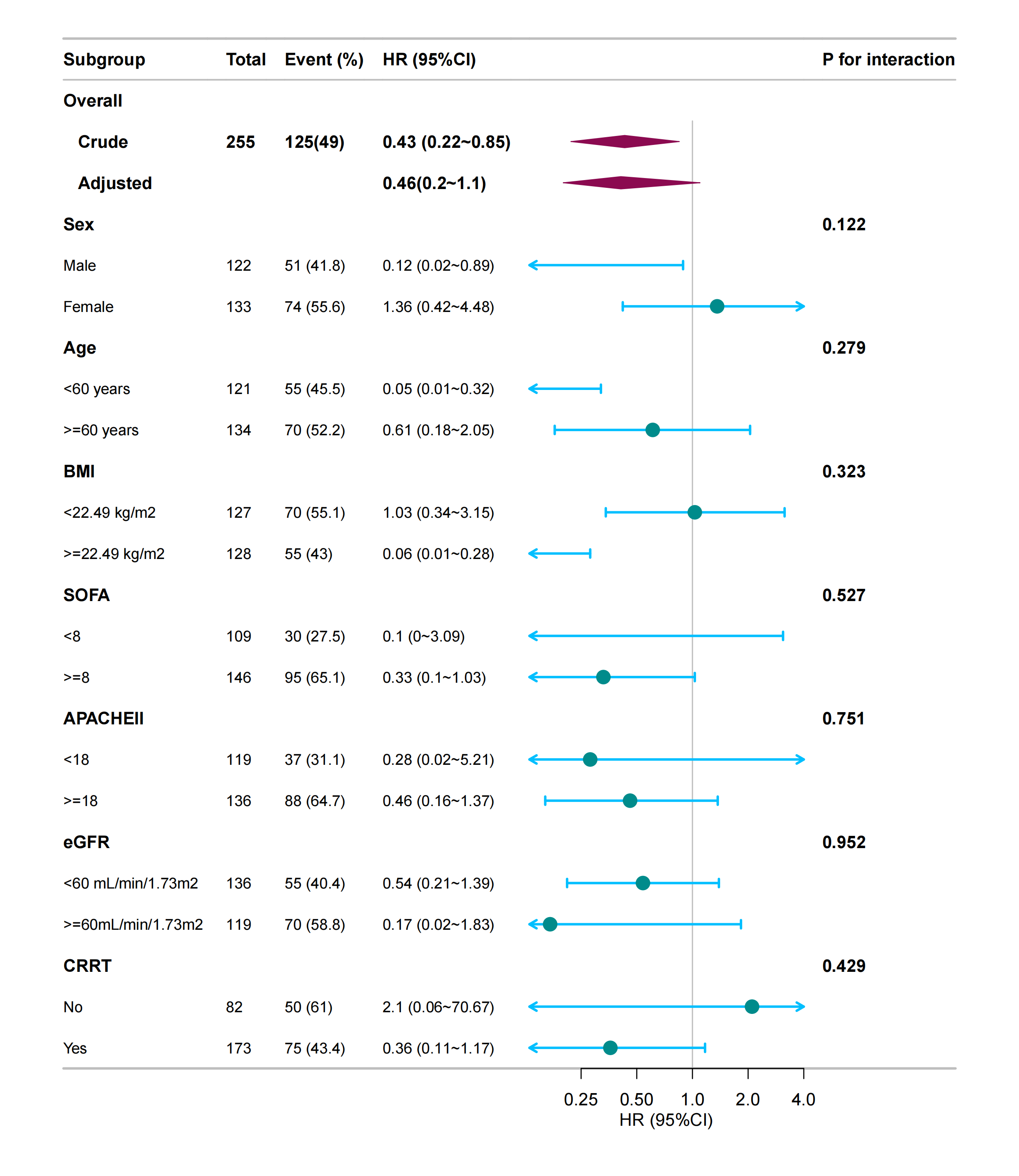


**Supplementary Figure S8. Stratified analyses of the association between Cr/CysC ratio and 28-day mortality status according to baseline characteristics in Sepsis database with acute kidney injury.**

Adjusted for age, sex, BMI,smoking ,drinking, infection,hypertension,Coronaryheart,diabetes,

arrhythmia,stroke,COPD,liver disease,cancer,Mechanical Ventilation,Vasoactive drug use,heart rate,MAP,PaO_2_/FiO_2_,Temperature,Lac,PLT,PCT,TBIL,ALB,PT,APTT,CRP,WBC, BUN,Uricacid,

β_2_-MG(model 5).
